# Fast Collective Hydrogen‐Bond Dynamics in Hexafluoroisopropanol Related to its Chemical Activity

**DOI:** 10.1002/anie.202416091

**Published:** 2024-11-18

**Authors:** Federico Caporaletti, Lucas Gunkel, M. Ángeles Fernández‐Ibáñez, Johannes Hunger, Sander Woutersen

**Affiliations:** ^1^ Van't Hoff Institute for Molecular Sciences University of Amsterdam Amsterdam The Netherlands; ^2^ Laboratory of Polymer and Soft Matter Dynamics Experimental Soft Matter and Thermal Physics (EST) Universitè libre de Bruxelles (ULB) Brussels Belgium; ^3^ Max Planck Institute for Polymer Research Mainz Germany

**Keywords:** fluorinated solvents, hydrogen bonds, IR spectroscopy, time-resolved spectroscopy, dielectric spectroscopy

## Abstract

Using fluorinated mono‐alcohols, in particular hexafluoro‐isopropanol (HFIP), as a solvent can enhance chemical reaction rates in a spectacular manner. Previous work has shown evidence that this enhancement is related to the hydrogen‐bond structure of these liquids. Here, we investigate the hydrogen‐bond dynamics of HFIP and compare it to that of its non‐fluorinated analog, isopropanol. Ultrafast infrared spectroscopy experiments show that the dynamics of individual hydrogen‐bonds is about twice as slow in HFIP as in isopropanol. Surprisingly, from dielectric spectroscopy we find the opposite behavior for the dynamics of hydrogen‐bonded clusters: collective rearrangements are 3 times faster in HFIP than in isopropanol. This difference indicates that the hydrogen‐bonded clusters in HFIP are smaller than in isopropanol. The differences in cluster size can be traced to changes in the hydrogen‐bond donor and acceptor strengths upon fluorination. The smaller cluster size can boost reaction rates in HFIP by increasing the concentration of reactive, terminal OH‐groups of the clusters, whereas the fast collective dynamics can increase the rate of formation of hydrogen‐bonds with the reactants. The longer lifetime of the individual hydrogen‐bonds in HFIP can enhance the stability of the hydrogen‐bonded clusters, and so increase the probability of reactant‐solvent hydrogen‐bonding.

## Graphical abstract

Hexafluoro‐isopropanol (HFIP) is a popular solvent strongly enhancing reaction rates. Its unique properties are thought to be connected to its hydrogen‐bond structure. Here we investigate the hydrogen‐bond dynamics of HFIP and its non‐fluorinated counterpart, isopropanol, by means of infrared and dielectric spectroscopy. We show that the smaller size and faster dynamics of hydrogen‐bonded clusters in HFIP play an important role in its superior solvent properties.

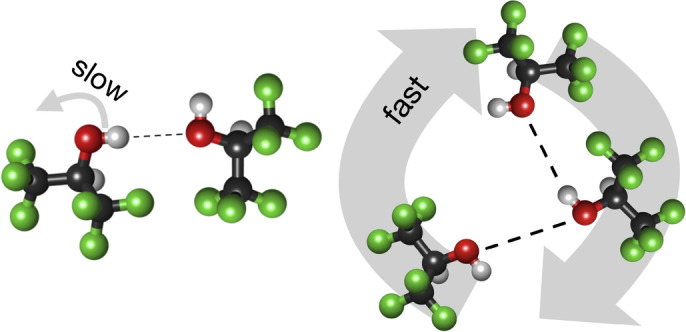

## Introduction

Fluorinated mono‐alcohols, in particular hexafluoro‐isopropanol (HFIP), can accelerate chemical reactions in spectacular ways, and many reactions take place only in these solvents.[^1^, ^2^, ^3^] In particular, when compared to its non‐fluorinated analogue, isopropanol, HFIP shows superior performance as a reaction medium.^[4]^ A well‐known example are metal‐catalyzed C−H functionalization reactions, where HFIP has become the most commonly used solvent.[^1^, ^2^, ^5^] HFIP also effectively stabilizes helical structures in proteins.[^2^, ^6^] Unraveling the molecular origins of the “booster effect”^[7]^ of fluorinated alcohols is an active field of research. The “booster effect” has been suggested to be due to activation of reactants via solvation/protonation of reactants or stabilization of ionic species/transition states,^[2]^ depending on the chemical reaction. More generally, irrespective of the specific reaction mechanism, all activation pathways via solvation and protonation are intimately related to the hydrogen‐bonding properties of HFIP. Based on combined ab initio calculations using the B3LYP density functional and NMRtitration and reactionkinetics experiments, Berkessel et al.^[7]^ have shown convincingly that the hydrogen‐bonding properties of HFIP play a crucial role. In particular, aggregation of HFIP molecules into hydrogen‐bonded clusters increases the hydrogen‐bond donor ability of the terminal hydroxyl proton, and reaction‐kinetics experiments show that 2–3 HFIP molecules are involved in the activation of the oxidant.^[7]^ Thus, hydrogen‐bonded clusters (rather than monomers) are crucial for the unique catalytic and solvolytic effects of HFIP.

Whereas the hydrogen‐bonding structure and energetics of HFIP have been studied experimentally and computationally,[^7^, ^8^, ^9^, ^10^, ^11^, ^12^, ^13^] little is known about the dynamics of the hydrogen‐bonds in fluorinated alcohols. Yet, the dynamics of the HFIP hydrogen‐bonds play a key role in theactivation of reactive intermediates via hydrogen‐bonding.^[14]^ Here, we explore the differences in hydrogen‐bond dynamics of HFIP and its non‐fluorinated counterpart (isopropanol) by combining ultrafast‐infrared and GHz‐dielectric spectroscopy. These methods provide complementary information on hydrogen‐bond dynamics:[^15^, ^16^] time‐resolved infrared spectroscopy probes the random orientational motion of individual OH groups,[^17^, ^18^, ^19^, ^20^] two‐dimensional infrared (2D‐IR) spectroscopy probes the distribution and fluctuations of the hydrogenbonds,[^20^, ^21^, ^22^, ^23^, ^24^, ^25^, ^26^, ^27^, ^28^, ^29^, ^30^, ^31^] and dielectric spectroscopy can track the dynamics of collective rearrangements, in particular the orientational random motion of hydrogen‐bonded aggregates.[^32^, ^33^, ^34^, ^35^] We find that intermolecular hydrogen‐bonds in liquid HFIP are weaker than in isopropanol. Surprisingly, whereas the hydrogen‐bond fluctuations and the reorientation of individual OH groups (as probed by time‐resolved infrared spectroscopy) are slower in HFIP than in isopropanol, the collective hydrogen‐bond dynamics (probed by dielectric spectroscopy) is significantly faster. Our results indicate that in HFIP the hydrogen‐bonded clusters are smaller and their collective rearrangements occur much faster than in isopropanol, whereas the individual hydrogen bonds, whose dynamics is reflected in individual molecular rearrangements, are longer lived. These differences can be explained by the subtle balance between hydrogen‐bond donor and acceptor strength: HFIP is a stronger hydrogen‐bond donor, and a weaker hydrogen bond acceptor, than isopropanol, resulting in shorter, but longer‐lived hydrogen‐bonded clusters in HFIP. The resulting larger number of free hydrogen‐bond donor groups in HFIP, which can donate stronger hydrogen bonds, can enhance reaction rates observed in HFIP.

## Results and Discussion

**Figure 1:**
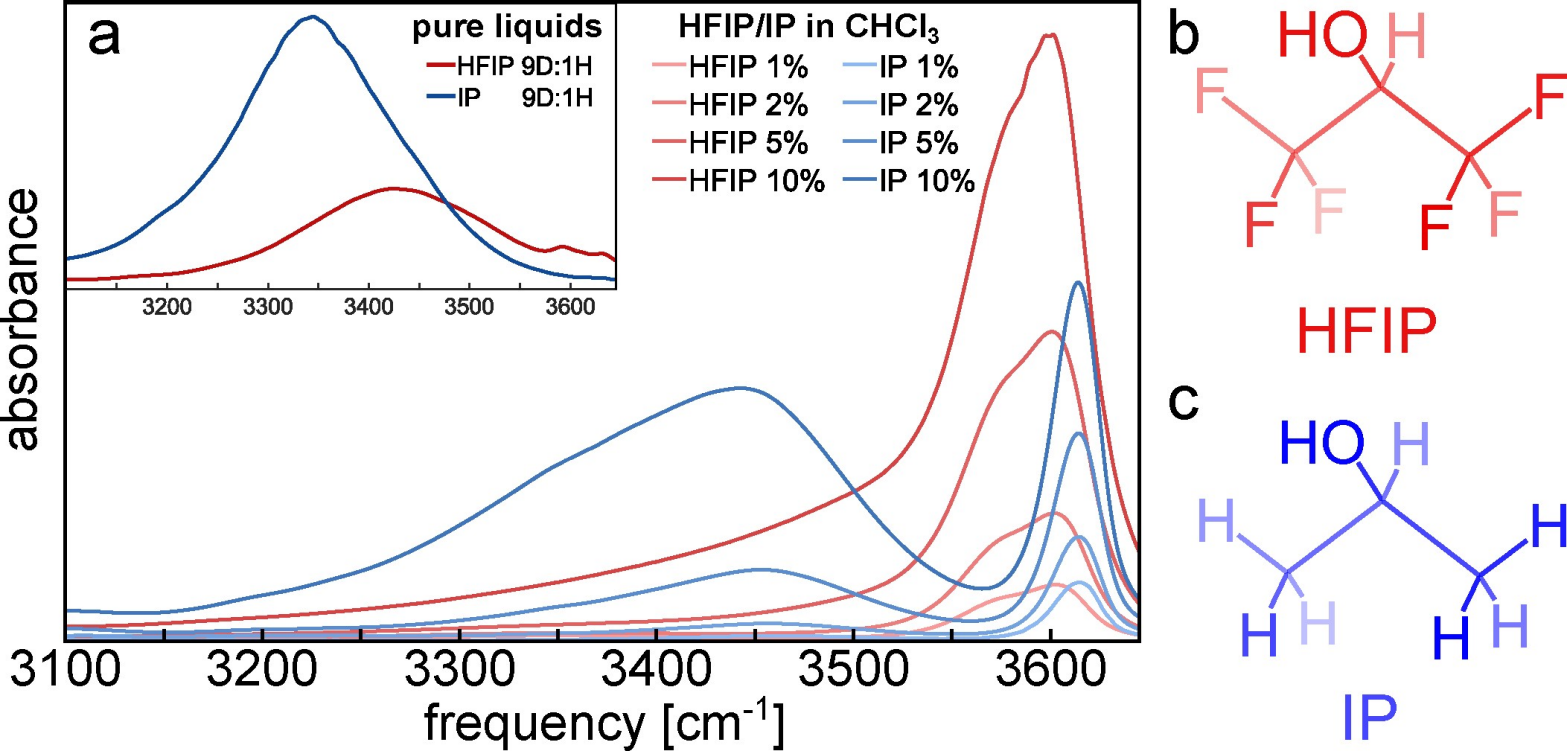
IR absorption spectra of increasing concentrations (in mol %) of HFIP (red) and isopropanol (blue) in chloroform. The spectra of the pure isotopically dilute (9D : 1H) alcohols are shown in the inset. Chemical structures of (b) hexafluoroisopropanol (HFIP) and (c) isopropanol (IP).

To investigate hydrogen bonding in HFIP and isopropanol we use the OH (or OD) stretching vibration of the alcohols, which is a sensitive probe of the hydrogen‐bond strength.^[36]^ To avoid excitonic‐coupling effects[^37^, ^38^] we study isotopically diluted liquids (HFIP‐OD in HFIP‐OH, or vice versa). In the inset of Figure 1 we show the OH‐stretch region of the IR spectra of isotope‐diluted HFIP and isopropanol (see Supporting Information for the OD‐stretch spectra). For HFIP, the spectrum exhibits weak peaks at ~3590 cm^−1^ and ~3630 cm^−1^ (OD: ~2650 cm^−1^ and ~2680 cm^−1^, see Figure S1), which are due to the antiperiplanar and synclinal conformers of the non‐hydrogen‐bonded molecules.[^8^, ^39^] These non‐hydrogen‐bonded OH groups in the liquid are somewhat (~30 cm^−1^) red‐shifted as compared to the gas phase,^[8]^ presumably due to weak interactions with the F atoms,[^40^, ^41^] but the OH‐stretch frequency difference between antiperiplanar and synclinal is very similar in the liquid and gas phase.^[8]^ The intense, red‐shifted OH‐stretching band centered at ~3350 cm^−1^ in isopropanol and at ~3420 cm^−1^ in HFIP is due to hydrogen‐bonded OH groups. Since the redshift with respect to the non‐hydrogen‐bonded frequency is proportional the hydrogen‐bond strength,^[36]^ the difference in hydrogen‐bonded OH‐stretch frequency between HFIP and isopropanol indicates that HFIP forms weaker hydrogen‐bonds with itself than does isopropanol. To confirm this, we record spectra of the two alcohols dissolved in chloroform at increasing concentrations (Figure 1). At low concentration, the spectra are dominated by the free OH peaks (at ~3620 cm^−1^ for isopropanol and ~3570 cm^−1^ and 3600 cm^−1^ for HFIP). With increasing concentration, isopropanol and HFIP show very different behaviour: for isopropanol, already at low concentrations a peak appears at ~3450 cm^−1^, which is due to hydrogen‐bonded alcohol clusters,^[42]^ while for HFIP the spectra are still dominated by the free OH peak even at the highest concentration. Thus, HFIP forms weaker hydrogen‐bonds with itself than does isopropanol. This may seem counter intuitive, since the “booster effect” of HFIP is believed to be related to its ability to form strong hydrogen‐bonds,[^7^, ^8^, ^9^, ^10^, ^11^, ^12^] but below we will see that there is in fact no contradiction.

To investigate the dynamics of individual hydrogen‐bonds, we track the reorientation of the OD (or OH) groups in HFIP and isopropanol in real time, using ultrafast infrared spectroscopy.[^15^, ^16^, ^22^, ^38^, ^43^, ^44^, ^45^] In the experiments, a short (~180 fs) infrared pulse excites (“tags”) the stretching mode of a small fraction of the OD (or OH) bonds of the liquid. The infrared light is polarized, and preferentially excites OD (or OH) bonds that are aligned parallel to the polarization of the excitation pulse. This results in an anisotropic distribution of excited OD (or OH) bonds, which can be characterized by the so‐called anisotropy parameter *R* (defined as $R=\left(\Delta A_{\left|\right|}-\Delta A_{⊥}\right)/\left(\Delta A_{\left|\right|}+2\Delta A_{⊥}\right)$ , where $\Delta A_{||,⊥}$ are the excitation‐induced absorption changes for light polarized parallel and perpendicular to the excitation polarization). The random motion of the OD groups randomizes the anisotropic distribution, leading to a decay of the anisotropy, and the decay of *R* directly mirrors the correlation function of the OD random orientational motion.[^15^, ^16^, ^22^] In fact, *R*(*t*) probes the dynamics of individual OH (or OD) groups.[^15^, ^16^]

**Figure 2:**
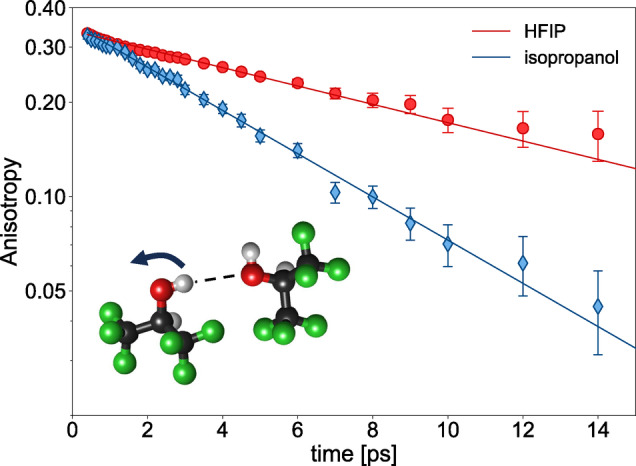
OD‐Stretch anisotropy decay of hexafluoro‐isopropanol (red circles) and isopropanol (cyan diamonds) measured at 2534 cm^−1^ and 2483 cm^−1^, respectively. The samples were isotopically diluted (1D:9H) to avoid coupling between the molecular oscillators. The solid lines show a least‐squares fit of a single exponential decay to the data. The inset shows the single‐molecule re‐orientation probed in these experiments. The error bars of *R*(*t*) correspond to ±1 standard deviation, and were estimated from the covariance matrix of the fitting procedure used to extract the re‐orientational function from the anisotropy measurement. More details on the extraction procedure can be found in the Supporting Information.

We obtain *R*(*t*) from the polarization‐dependent absorption changes of the hydrogen‐bonded OH (or OD) groups after correcting the data for a small thermal contribution using a procedure similar to that of Ref. [46] (see Supporting Information for the details, Figures S2–S6). Figure 2 shows the anisotropy decay of the OD groups of HFIP (red circles) and isopropanol (cyan diamonds), as observed in OD/OH dilute isotopic mixtures (the arrows in Figure S1 indicate the frequencies at which the anisotropy decay was measured). The time dependence of *R*(*t*) can be well described using a single‐exponential decay, and we find that the decay time is approximatelytwice as fast in isopropanol than in HFIP (a similar result is obtained for isotopically diluted HFIP‐OH in HFIP‐OD, see Figure S7). Thus, despite the weaker hydrogen‐bonds in HFIP (see Figure 1), individual OD groups in HFIP reorient slower than in isopropanol.

The decay of *R*(*t*) for hydrogen‐bonded liquids can occur via different molecular mechanisms. At short timescales (<0.5 ps) inertia‐limited motions, such as librations, can result in a loss of orientational correlation.^[47]^ Our data however show no evidence for a marked, fast *R*(*t*) decay at short times. On longer timescales, molecular reorientation via angular jumps during which OH‐groups exchange hydrogen‐bond acceptors together with the rotation of intact hydrogen‐bonds—the so‐called frame orientation—give rise to the decay of the orientational memory.^[47]^ For water, the loss of orientational correlation due to the jumps dominates the decay of *R*(*t*) due to the large jump angle, while the frame orientation is somewhat slower. For alcohols, the orientation of intact hydrogen‐bonds is even slower than in water (see discussion of the dielectric spectra below) and contributions due to the slower frame orientation to the *R*(*t*) decay are very weak.^[19]^ Moreover, due to the fast decay of the vibrational excitation for our samples on a ~1 ps timescale, which limits our accessible time window for recording the *R*(*t*) decays, the ultrafast infrared experiment is only weakly sensitive to slow dynamics. Hence, the observed decay of *R* is dominated by hydrogen‐bond exchange dynamics. In this case, the reorientation dynamics are limited by the availability of potential hydrogen‐bond acceptors to form a new hydrogen‐bond after hydrogen‐bond breaking, that is, the density of OH groups.[^47^, ^48^, ^49^] Indeed, liquid HFIP has a lower hydroxyl‐group density (5.7 OH‐groups/nm^3^) than ispropanol (7.9 OH‐groups/nm^3^), which can explain the slower hydrogen‐bond dynamics in the probed time‐window. Similarly, steric congestion can also play an important role in slowing down exchange dynamics in HFIP, similarly to what has been observed in MD simulations of other monohydroxyl alcohols.^[48]^ To resolve the exact molecular‐level details underlying the detected orientational dynamics of the OH groups, molecular dynamics simulations would be required, and we hope that our results will stimulate work in this direction. Irrespective of the exact reorientation mechanism, our results indicate that individual hydrogen‐bonds in HFIP, at the ps timescale, are less dynamic than those in isopropanol.

**Figure 3:**
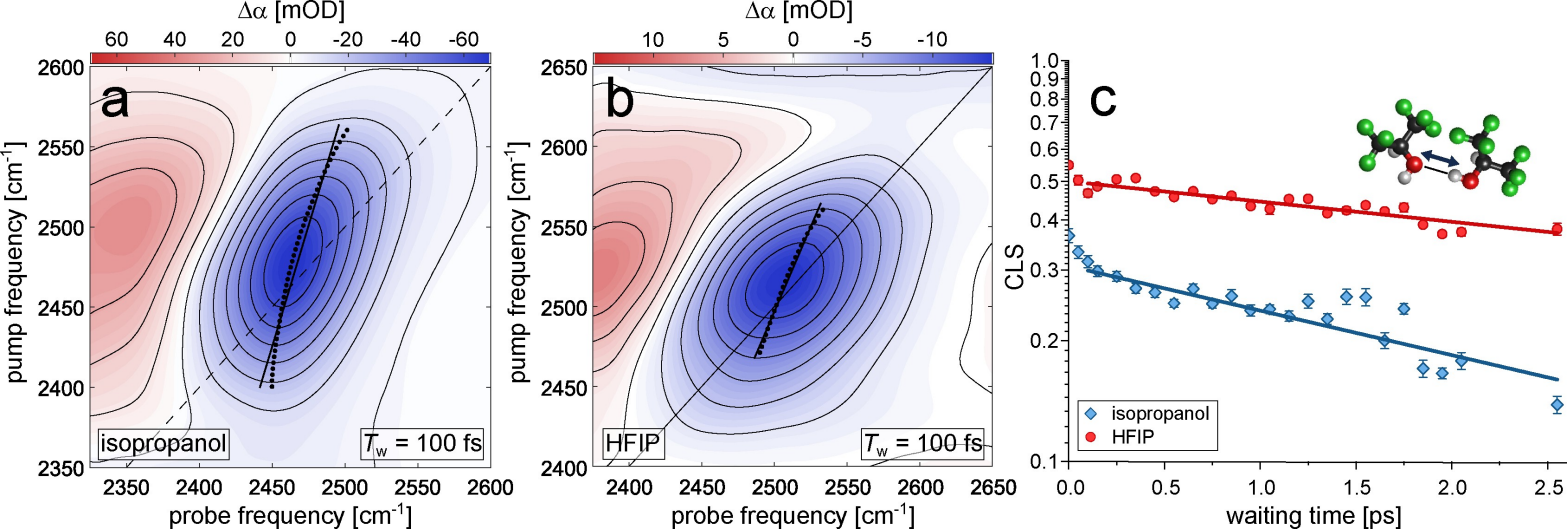
2D‐IR spectra (parallel pulse polarizations) at 100 fs for the OD‐stretch band of a) isopropanol and b) HFIP (1D:9H). The black symbols show the minima of the bleaching signal at a given excitation frequencies together with a linear fit – the center line (solid). c) Time‐dependent center line slope (CLS) for isopropanol (blue) and HFIP (red). The CLSs are the inverse slopes of the lines shown in panels a) and b). Symbols show experimental data and solid lines mono‐exponential fits.Error bars are the standard errors obtained from linear regression of the center line points. The inset illustrates the hydrogen‐bond fluctuations probed by these experiments.

We further investigate the dynamics of individual hydrogen‐bonds using two‐dimensional infrared (2D‐IR) spectroscopy^[22]^ (the description of the experimental setup is given in Refs. [49, 50]). In these pump‐probe experiments, we vary the infrared exciting and probing frequencies, and measure the absorption change as a function of both these frequencies.^[22]^ The tilt of the contours (the “center line slope” or CLS) in the 2D‐IR spectrum (Figure 3a, b; see Figure S8 for other waiting times) shows to what extent the response depends on the excitation frequency, and the time dependence of this slope (Figure 3c) mirrors the correlation function of the fluctuations in the OD‐stretch frequency,[^21^, ^22^] and hence of the fluctuations in the hydrogen‐bond length.^[21]^ We find that the slope at time zero is smaller in isopropanol than in HFIP (0.37 vs 0.55), which implies a more heterogeneous hydrogen‐bond distribution in HFIP as compared to isopropanol.^[22]^ The decay of the slope in the two liquids (Figure 3c) shows that the OD‐stretch frequency fluctuations are somewhat slower in HFIP than in isopropanol (see Table 1 for the time constants; the uncertainties in this table are an underestimate of the actual uncertainties, since the statistically determined error bars on our data points are much smaller than the actual errors). These frequency fluctuations are due to breaking and re‐formation of hydrogen bonds (required for rotation of the OD bonds probed in the experiment of Figure 2) and fluctuations in hydrogen‐bond length and angle.^[20]^ Thus, the CLS dynamics confirm that the hydrogen bonds in HFIP are less dynamic than in isopropanol.

**Table 1:** Relaxation times τ_j_ obtained from least‐squares fits to the time‐resolved infrared, center line slopes, and dielectric‐spectroscopy data. See Supporting Information for details of the data analysis.

	TRIR	2D‐IR	Dielectric spectroscopy		
	τ_or_/ps	τ_CLS_/ps	τ_1_/ps	τ_2_/ps	τ_3_/ps
isopropanol	7±1	3.9±0.4	408±5	12.6±0.8	1.7±0.2
HFIP	14±1	8.6±0.7	140±11	17.5±2.7	2.4±0.3

To probe the collective dynamics of hydrogen‐bonded clusters in HFIP and isopropanol, we use dielectric spectroscopy. With this technique the molecular motion in response to an external, alternating electric field is monitored and the spectra are sensitive to both single and, especially, collective hydrogen‐bond dynamics, thus providing a complementary view on hydrogen‐bond structure and dynamics.^[15]^ It is important to stress that the collective dynamics of supramolecular structures in alcohols is expected to be significantly slower than the individual re‐orientation of HFIP molecules, as cooperative re‐arrangements of more molecules are required.

**Figure 4:**
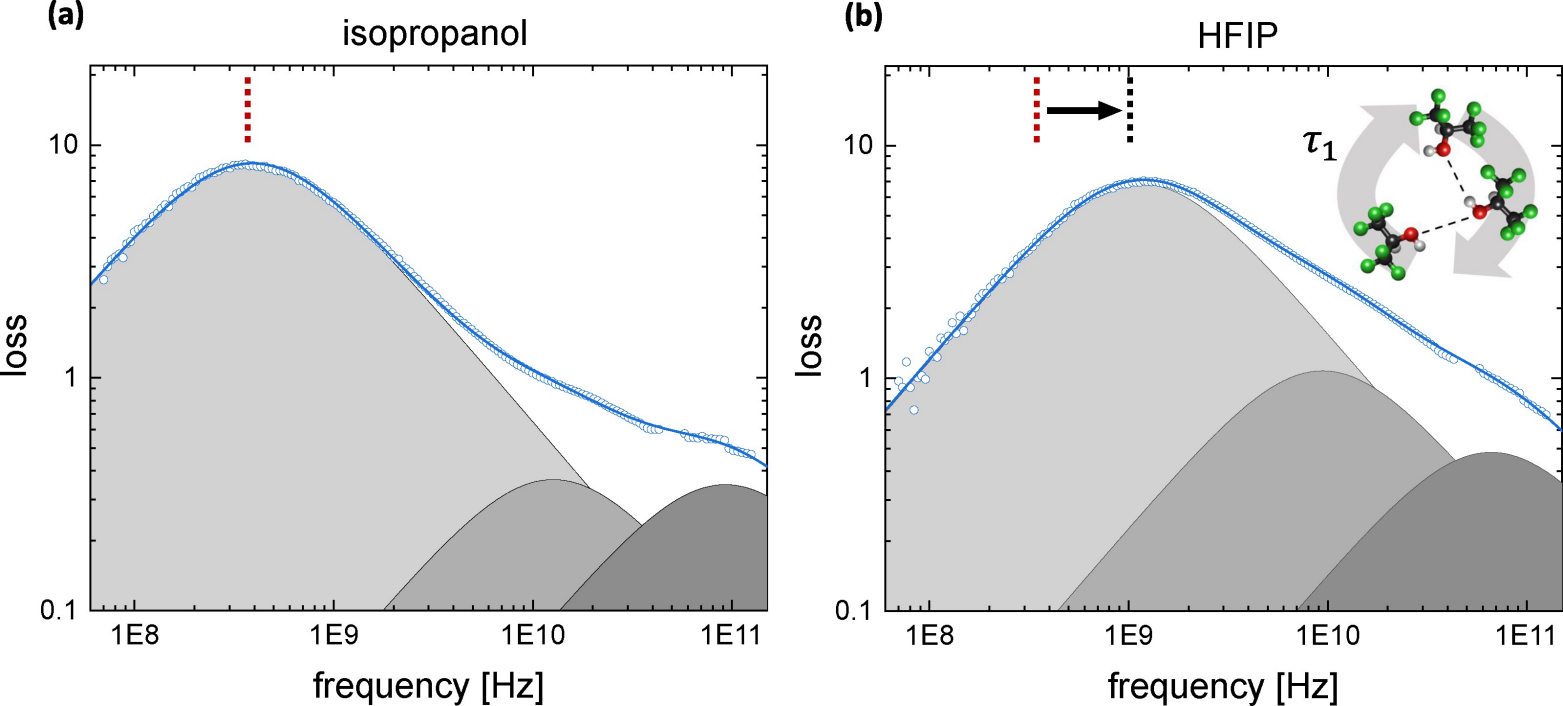
Dielectric‐loss spectra of (a) isopropanol and (b) HFIP. Symbols show experimental data, solid lines show fits of a combination of three Debye peaks to the data (see Supporting Information for details). Shaded areas show the contributions of the individual Debye peaks to the spectrum. The minor offsets in the data at frequencies >50 GHz are of instrumental origin due to the use of different coaxial probes and phase instabilities of the external frequency converter (see SI). The inset schematically illustrates the type of process probed by the low‐frequency peak in these experiments.

The random orientational motions of electric dipoles (either due to motion of individual molecules or of clusters) give rise to broad peaks in the dielectric‐loss spectra 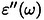 of a liquid, where the peak frequencies are determined by the characteristic time scales of these motions.^[51]^ In mono‐alcohols, the dielectric spectra in the MHz to GHz frequency region generally contain three distinct peaks. The two peaks at high frequencies are predominantly due to the fast rearrangements of individual molecules.^[52]^ Although there are differences in the detailed interpretation[^34^, ^35^, ^53^, ^54^, ^55^, ^56^, ^57^, ^58^, ^59^, ^60^, ^61^], the peak at low frequency is usually associated with the slower, collective rearrangements or dipolar cross‐correlations arising from supramolecular structures: as the external electric field induces fluctuations of all molecules at the same time, the motion of an individual dipolar molecule is affected by both, the varying external field and the motion of dipoles in its direct vicinity (dipole‐dipole correlations). As such, the lower‐frequency dielectric peak mirrors the average size of hydrogen‐bonded clusters (the lower its frequency, the larger the average cluster size/ dipole correlations).^[34]^ Figure 4 shows the dielectric spectra of HFIP and isopropanol. Both spectra can be well described by a combination of three (Debye‐type) peaks, and from a least‐squares‐fit analysis (shown as the curves in Figure 4; see Supporting Information and Refs. [62–66] for details) we obtain the time constants and amplitudes associated with each of the three peaks. The time constants are listed in Table 1 (see Supporting Information for the amplitudes). The dynamics on fast time scales, characterized by time constants *τ*_2_ and *τ*_3_, show the same trend as we observed in the time‐resolved infrared experiments: both these relaxation times are shorter in isopropanol than in HFIP, indicating that individual hydrogen‐bond rearrangements occur slower in HFIP than in isopropanol. The *τ*_2_ and *τ*_or_ values are of similar magnitude, which suggests non‐diffusive dynamics (in the limit of diffusive reorientational dynamics, the dielectric spectroscopy time should be ≈3 times slower than the time‐resolved infrared time).^[67]^ Such non‐diffusive dynamics could arise from restricted dynamics, where the hydrogen‐bonded structure imposes constraints on the angular degrees of freedom of the molecules, impeding reorientations with small angular increments.

Whereas the time‐resolved infrared and dielectric‐spectroscopy experiments both show that individual hydrogen‐bond rearrangements are slower in HFIP than in isopropanol, we find the opposite behavior for the collective dynamics: the most intense, low‐frequency peak in the dielectric spectrum is at a much higher frequency for HFIP than for isopropanol (light‐grey peak in Figure 4), and the associated time constant *τ*_1_ is nearly three times shorter for HFIP (Table 1). The shorter *τ*_1_ time for the collective motion of HFIP clusters, as opposed to the slower dynamics of individual hydrogen‐bonds in HFIP as compared to isopropanol, implies less cooperativity in the observed lower‐frequency dielectric relaxation. These reduced correlations can be explained by significantly smaller hydrogen‐bonded aggregates in HFIP than in isopropanol. The smaller cluster size is in line with findings for fluorinated and non‐fluorinated tert‐butanol^[68]^ and is consistent with the higher strain (and hence, lower barrier for opening OH⋅⋅⋅O hydrogen‐bonds) in small ring‐like HFIP clusters observed in the gas phase.^[13]^ This difference in cluster size is confirmed by the larger spectral heterogeneity of the OD‐stretch mode in the 2D‐IR experiments (small aggregates have a larger proportion of terminal hydrogen‐bonds, which differ in strength from the hydrogen‐bonds in the interior of the aggregate, and this translates into a broader distribution of OD‐stretch frequencies).[^20^, ^42^, ^69^, ^70^] Similarly, more heterogeneous hydrogen‐bonds giving rise to a broader distribution of vibrational lifetimes (Figure S9),^[70]^ and also the enhanced number of non‐bonded OD groups in HFIP (Figure 1) support reduced cluster sizes in HFIP.

## Conclusions

**Figure 5:**
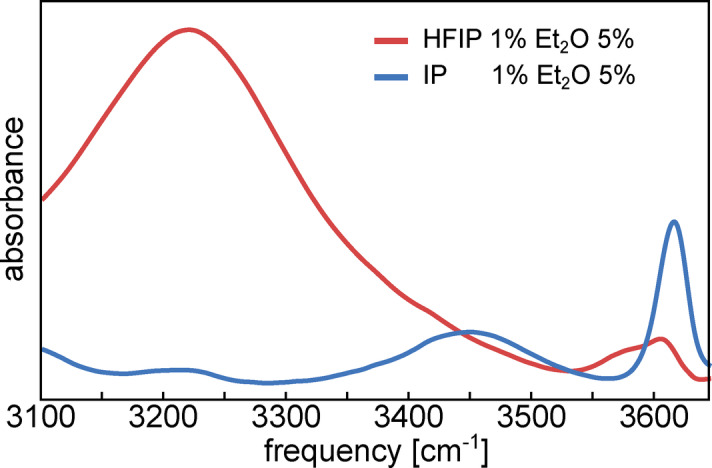
IR absorption spectra of mixed solutions in CHCl_3_, containing 5 mol % Et_2_O and 1 mol % HFIP or isopropanol. Note the reverse order of the OH⋅⋅⋅Et_2_ hydrogen‐bonded OH‐stretch peaks of HFIP and isopropanol as compared to hydrogen‐bonded peaks in the pure liquids, shown in the inset of Figure 1.

Summarizing, our measurements indicate that compared to isopropanol, HFIP consists of smaller, faster‐moving hydrogen‐bonded clusters, in which the internal, somewhat weaker hydrogen‐bonds are less dynamic. How are these differences between HFIP and isopropanol related to the difference in chemical activity of these two liquids? The weaker hydrogen bonds in HFIP as compared to isopropanol may at first sight appear counter‐intuitive, as fluorination of alcohols enhances the hydrogen‐bond donor strength and, often equivalent, acidity.[^71^, ^72^, ^73^] However, fluorination also makes alcohols poorer hydrogen‐bond acceptors.[^72^, ^73^] Indeed, density functional theory calculations of hydrogen‐bonded dimers suggest that the interaction energy of 2 HFIPs and 2 isopropanols are comparable (see Figure S10 while technical details are available in the Supporting Information and in Refs. [74–83]), whereas HFIP donating a hydrogen bond to isopropanol is energetically much more favorable than isopropanol donating a hydrogen bond to HFIP (Figure S10). This notion is in line with previous NMR‐titration studies by Berkessel et al.).^[7]^ Berkessel et al. have shown convincing evidence that the catalytic activity of HFIP is caused by hydrogen‐bonded clusters in this solvent. Interestingly, their calculations show that the hydrogen‐bond donor capacity of a terminal HFIP molecule in a hydrogen‐bonded cluster increases with cluster size, but that this effect already levels off at a cluster size of 3; and their chemical‐kinetics experiments show that aggregates of only 2–3 monomers are responsible for the activation of the oxidant.^[7]^ Our results suggest that HFIP consists of many small hydrogen‐bonded clusters, which are sufficiently large and have a sufficiently long lifetime to efficiently boost chemical conversions, yet sufficiently small (smaller than in isopropanol) to posses a large number of available active terminal sites which can donate a hydrogen bond to a reactant molecule. These active terminal OH groups of HFIP can efficiently enhance chemical reaction rates by activating reactants via strong hydrogen‐bond donation. The hydrogen‐bonds donated to reactants by HFIP are in fact stronger than those donated by isopropanol, as we demonstrate for the hydrogen‐bonds between the alcohols and the model reactant diethylether in chloroform solution, see Figure 5. The infrared spectra of 1 mol % HFIP or isopropanol are dominated by the non‐hydrogen‐bonded OH stretching bands at ~3600 cm^−1^. Upon addition of the hydrogen‐bond acceptor diethylether, the OH stretching bands of hydrogen‐bonded OH⋅⋅⋅OEt_2_ groups emerge at lower frequency: at ~3450 cm^−1^ for isopropanol and ~3200 cm^−1^ for HFIP, where the reversal in the hydrogen‐bonded peak order with respect to Figure 1 (inset) should be noted. This marked difference in hydrogen‐bonded OH‐stretch frequencies shows that HFIP forms much stronger hydrogen bonds to ether than does isopropanol. The difference in hydrogen‐bond strength is further evidenced by the spectral amplitudes: non‐bonded OH groups nearly fully vanish at 10 mol % diethylether for HFIP, while a large fraction of non‐bonded OH groups are present for isopropanol (Figure S11a). To quantify the binding strengths, we determine the degree of association from the spectral amplitudes as a function of the diethylether concentration (Figure S11b–c, see Supporting Information for details). The data in Figure S11c can be described by bimolecular association^[84]^ with association constants $K_{\mathrm{ass}}\left(\mathrm{IP}\right)$ =0.8 $\frac{L}{\mathrm{mol}}$ and $K_{\mathrm{ass}}\left(\mathrm{HFIP}\right)$ =3.9 $\frac{L}{\mathrm{mol}}$ , qualitatively consistent with DFT calculations (Figure S10). Our experiments suggest about two times stronger hydrogen‐bonding of HFIP to diethylether as compared to isopropanol.Our spectroscopic results demonstrate that this subtle balance between the donor and acceptor strength results in less extended hydrogen bonding in HFIP. As a consequence, (1) at ambient temperature the concentration of terminal (i.e., reactive, hydrogen‐bond donating)^[7]^ OH groups is larger in neat HFIP than in isopropanol; (2) due to the faster collective dynamics (i.e., the re‐orientation of aggregates as a whole), these reactive terminal OH groups have a higher frequency of encounters with the reactant molecules; (3) the hydrogen‐bonds within HFIP clusters are less dynamic (as evidenced from the slower OH‐reorientation and CLS dynamics); finally, (4) the terminal OH groups of the HFIP clusters can donate strong hydrogen‐bonds to the reactants. The latter can be seen from the linear absorption spectra and the DFT‐calculated hydrogen‐bond strengths between the alcohols and diethylether, which serves as a proxy for a large class of reactants.^[7]^ Thus, our dynamical results complement the structural booster effects reported by Berkessel et al.,^[7]^ and shed new light on the enhanced reactivity in HFIP as compared to conventional alcohols such as isopropanol.

## Conflict of Interests

The authors declare no conflict of interest.

1

## Supporting information

As a service to our authors and readers, this journal provides supporting information supplied by the authors. Such materials are peer reviewed and may be re‐organized for online delivery, but are not copy‐edited or typeset. Technical support issues arising from supporting information (other than missing files) should be addressed to the authors.

Supporting Information

## Data Availability

The data that support the findings of this study are available from the corresponding author upon reasonable request.
