# Supplementary material for: Fast Collective Hydrogen‐Bond Dynamics in Hexafluoroisopropanol Related to its Chemical Activity
Source: Angew Chem Int Ed Engl. 2024 Nov 18;63(52):e202416091. doi: 10.1002/anie.202416091 (PMC11656151; doi:10.1002/anie.202416091)
Supplement: Supplementary file 1 — Supporting Information [file ANIE-63-e202416091-s001.pdf]

Angewandte Chemie
Eine Zeitschrift der Gesellschaft Deutscher Chemiker

Supporting Information

Fast Collective Hydrogen-Bond Dynamics in Hexafluoroisopropanol Related to its Chemical Activity

F. Caporaletti, L. Gunkel*, M. Á. Fernández-Ibáñez, J. Hunger, S. Woutersen*

Supplementary Information: Fast Collective Hydrogen-Bond Dynamics in Hexafluoroisopropanol Related to its Chemical Activity

Federico Caporaletti,[*,†,‡,§] Lucas Gunkel,[¶,§] María Ángeles Fernández-Ibáñez,[†] Johannes Hunger,[*,¶] and Sander Woutersen[*,†]

† *Van 't Hoff Institute for Molecular Sciences, University of Amsterdam, Amsterdam, The Netherlands*

‡ *Laboratory of Polymer and Soft Matter Dynamics, Experimental Soft Matter and Thermal Physics (EST), Université libre de Bruxelles (ULB), Brussels 1050, Belgium*

¶ *Max Planck Institute for Polymer Research, Mainz, Germany*

§ *Contributed equally to this work*

E-mail: federico.caporaletti@ulb.be; hunger@mpip-mainz.mpg.de; s.woutersen@uva.nl

Materials and Methods

Materials

1,1,1,3,3,3-hexafluoroisopropanol (HFIP, spectroscopy grade, $> 99.9\,\%$, Fisher Scientific) and isopropanol (Optima LC/MS Grade, $> 99.9\,\%$, Fisher Scientific). Deuterated HFIP-d2 and isopropanol were purchased from Eurisotop (99,00% D, water content $< 0.3\%$). Diethylether ($\geq 99.5\%$) was purchased from Carl Roth and Chloroform ($99+\%$) was purchased by Acros

Organics. All chemicals were used without further purification. Deuterated samples were handled under an inert Argon atmosphere.

Linear Infrared spectroscopy

Transmission infrared spectra were recorded using a Bruker Vertex 70 IR spectrometer, with a resolution of 4 cm^{-1} between 400 cm^{-1} to 4000 cm^{-1}. During measurement the spectrometer was flushed with dried air. Linear spectra of isotopically dilute alcohols were measured using a 50μm spacer and measurements in chloroform were conducted using a 200μm spacer.

Infrared pump-probe spectroscopy

Mid-IR vibrational pump-probe spectroscopy was used to measure the decay of the vibrational anisotropy of the OD-stretch vibration of isotoped-diluted solutions of HFIP and isopropanol. The femtosecond IR pulses (5 μJ) used in the experiments were characterized by a center frequency of 2500 cm^{-1}, duration ~180 fs (FWHM) and spectral width of 150 cm^{-1} (FWHM) and were generated by a series of nonlinear frequency-conversion processes pumped by a commercial Ti:sapphire regenerative amplifier (for details see Ref. 1). The probe and reference beams were generated by splitting off a small portion (a few %) of the IR light with a wedged CaF_2 window, while the transmitted light provided the pump beam. The measurements were performed on isotopically diluted samples (D/H=0.1) to avoid coupling between molecular oscillators.[2–4] The bandwidth of the pump and probe beams was $\sim$ 150cm^{-1}, large enough to avoid the influence of spectral diffusion on the anistropy measurements. The liquid samples were loaded into 200μm thick cells equipped with CaF_2 windows.

Two-dimensional Infrared (2D-IR) spectroscopy

The setup is described in more detail in our previous work.[5,6] Briefly, we used 800 nm laser pulses (5.6 W, 35 fs, 1 kHz) from a regenerative amplifier (Coherent Astrella) to generate 4000 nm IR pulses (25 μJ, 400 nm full width at half maximum) with an optical parametric amplifier (Topas Prime, Coherent) and noncollinear difference frequency generation (NDFG). The IR pulses are guided into a commercial 2D-IR spectrometer (2D Quick IR, Phasetech Inc.). A small fraction of the IR pulses is split off as the probe pulse. We generated pump pulse pairs, delayed by 0 fs to 700 fs at increments of 35 fs, using a pulse shaper based on an acousto-optic modulator (AOM). The pump frequency was resolved by Fourier transformation of these time-domain data. A rotating frame at 2300 cm^{-1} was used to reduce the measuring time. Before Fourier transformation, the data was zero padded to 128 data points and a Hamming window was applied. Pump and probe pulses were focused and overlapped in the samples, which were held between CaF_2 windows separated by 100 μm teflon spacers. We use a pump probe geometry yielding absorptive 2D-IR spectra.[7] The probe frequency was resolved in the frequency domain by dispersing the probe pulses onto a 128 x 128 mercury cadmium telluride (MCT) array detector. All spectra were recorded with parallel polarized excitation and detection pulses. For better comparability, signals were Fourier-filtered analogously along the probe axis.

Dielectric relaxation spectroscopy

Complex permittivity spectra $\hat{\varepsilon}(\nu) = \varepsilon'(\nu) - \mathrm{i}\varepsilon''(\nu)$, where ε' is the dielectric permittivity and ε'' the dielectric loss, were measured as function of field frequency, ν, using a frequency domain reflectometer using flanged open-ended coaxial probes.[8–10] Frequencies at $0.07 \leq \nu/\mathrm{GHz} \leq 0.52$ were covered using coaxial probes made from SMA hermetic feedthroughs and frequencies $0.53 \leq \nu/\mathrm{GHz} \leq 43$ using coaxial probes based on 1.85 mm feedthroughs connected to a vector network analyzer (VNA, Anritsu MS4647A). Spectra at $57 \leq \nu/\mathrm{GHz} \leq 125$ were recorded using a 1 mm connector coaxial probe connected to an external frequency

converter (Anritsu 3744A mmW) in combination with the VNA.[11] The reflection coefficient at the coaxial-probe/sample interface was calibrated using air, conductive silver paint, and water as references.[12] Spectra were recorded from 5 mL of the sample in a glass vial in contact with the coaxial probes.

Density functional theory calculations

To estimate the differences in hydrogen-bond strengths formed by the two alcohols, we determined interaction energies for HFIP and isopropanol donating hydrogen-bonds to HFIP, isopropanol, and diethylether using density functional theory (DFT) calculations with the ORCA program package (version 5.0.4).[13] All calculations were performed at the B3LYP,[14–17] 6-311++G(d,p)[18] level of theory. The solvent was accounted for using the CPCM solvation model[19–22] using chloroform as solvent. To determine the interaction energies we optimized the geometry of the dimers. Subsequently, we displaced the two molecules and performed a second geometry optimization with the distance of the two oxygen atoms (the O of the hydrogen-bond donating OH group and the hydrogen-bond accepting O) constrained to 30 Å. The interaction energy was taken as the difference in the single point energies of both geometry optimizations, which includes electronic and solvation contributions.

Fit parameters obtained from the dielectric spectra

The dielectric spectra can be well described by a combination of three (Debye-type) peaks, and from a least-squares-fit analysis using the fit function

$$\hat{\varepsilon}(\nu) = \sum_{j=1}^{3} \frac{\Delta\varepsilon_j}{1 + i2\pi\nu\tau_j} + \varepsilon_\infty, \tag{S1}$$

where $\Delta\varepsilon_j$ are the relaxation strengths, τ_j the relaxation times, and ε_∞ is the high frequency limit of the permittivity. All fits were performed by minimizing the sum of the squared

deviations on a logarithmic scale and error bars were obtained from the standard deviations of fitting at least six independently recorded spectra. The obtained fit parameters are listed in Table S1. While τ_j identifies the characteristic timescale of a relaxation mode, $\Delta\varepsilon_j$ is proportional to the number of dipoles reorientating at τ_j. The obtained fitting parameters are reported in Table S1.

Table S1: Fit parameters obtained from fitting eq. 1 to the experimental permittivity spectra: relaxation amplitudes $\Delta\varepsilon_j$; relaxation times τ_j, and infinite frequency permittivity, ε_∞.

	$\Delta\varepsilon_1$	τ_1 / ps	$\Delta\varepsilon_2$	τ_2 / ps	$\Delta\varepsilon_3$	τ_3 / ps	ε_∞
isopropanol	16.65 ± 0.06	408 ± 5	0.75 ± 0.06	12.6 ± 0.8	0.70 ± 0.05	1.7 ± 0.2	2.43 ± 0.03
HFIP	13.66 ± 0.36	140 ± 11	2.2 ± 0.2	17.5 ± 2.7	0.98 ± 0.13	2.4 ± 0.3	2.17 ± 0.05

Infrared absorption spectra of HFIP and isopropanol

Figure S1 shows the linear absorption spectra of OD/OH isotope-diluted isopropanol and HFIP. The OD-stretching band of HFIP is located at a higher frequency with respect to isopropanol. The small peaks at high frequencies have been assigned previously to free OH-groups[23,24] of different conformers. The arrows indicate the frequencies at which we measured the single-molecule reorientation dynamics of HFIP and isopropanol.

Analysis of transient absorption spectra to obtain the anisotropy

The rotational anisotropy is extracted starting from the parallel and perpendicular components of the transient absorption signal $\Delta\alpha_{||}$ and $\Delta\alpha_\perp$

$$R = \frac{\Delta\alpha_{||} - \Delta\alpha_\perp}{\Delta\alpha_{||} + 2\Delta\alpha_\perp}\,. \tag{S2}$$

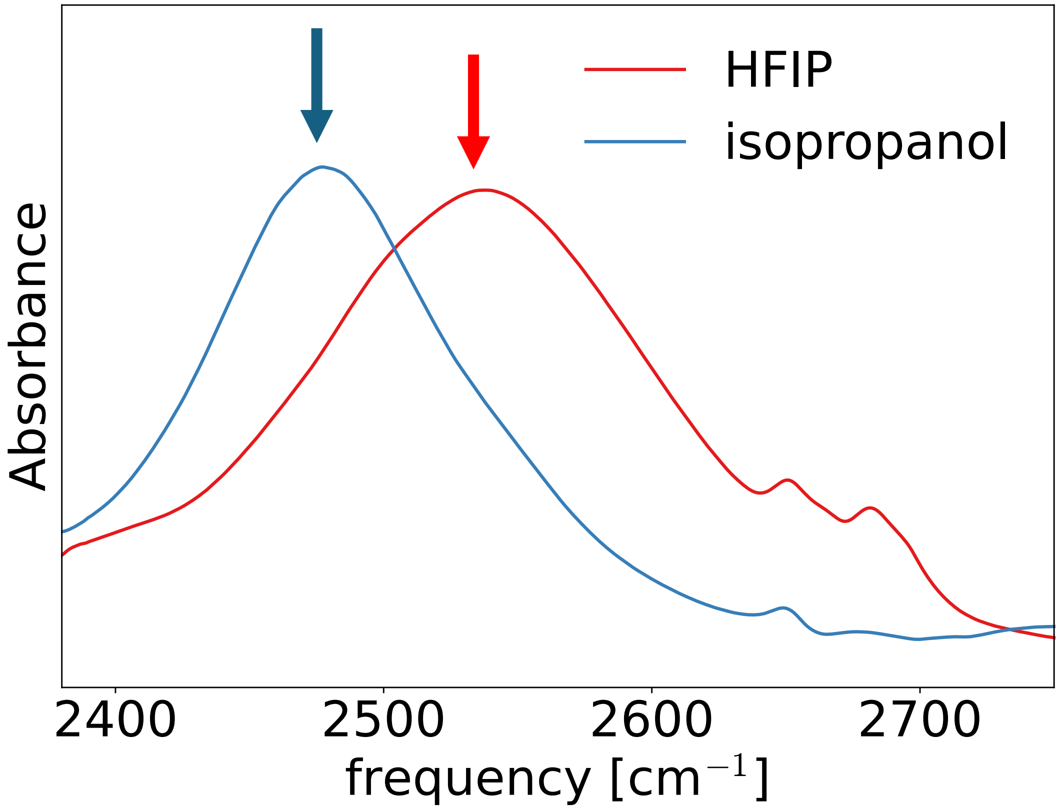

Figure S1: IR absorption spectra of isotope diluted (OD/OH=0.1) HFIP (red solid line) and isopropanol (blue solid line). The arrows indicate the central frequency of the broad-band pulses used to measure the anisotropy decay.

The denominator of R is the isotropic signal

$$\Delta\alpha_{iso} = \frac{\Delta\alpha_{||} + 2\Delta\alpha_{\perp}}{3}\,. \tag{S3}$$

$\Delta\alpha_{iso}$ is independent of molecular reorientation and purely reflects the relaxation of the vibrational excitation and the subsequent thermalization. Heating effects produced by the pump pulse give rise to an additional small contribution to the transient absorption, and as a consequence the anisotropy cannot be obtained directly from $\Delta\alpha_{||,\perp}$, but only after subtracting such thermal contribution, a correction procedure that is described in detail in refs. 25,26

Vibrational relaxation

The vibrational dynamics of the excited OD vibration of isotopically diluted mixtures of both HFIP and isopropanol has been modeled as a multi-step decay process, see Fig. S2(a). The first excited state decays first to an intermediate state, and finally, passing through an additional state, relaxes to a hot ground state. Modeling the data with only 1 intermediate state could not reproduce the data well. The time-dependent populations in this model are determined by the differential equations:

$$\frac{d}{dt}N_i(t) = k_{ij}N_j(t) \tag{S4}$$

where $N_i(t)$ is the population, t is the delay between the pump and probe pulse, and k_{ij} are the relaxation rates defined by the kinetic model, in this case given by

$$k_{ij} = \begin{bmatrix} -k_1 & 0 & 0 & 0 \\ +k_1 & -k_2 & 0 & 0 \\ 0 & +k_2 & -k_3 & 0 \\ 0 & 0 & +k_3 & 0 \end{bmatrix} \tag{S5}$$

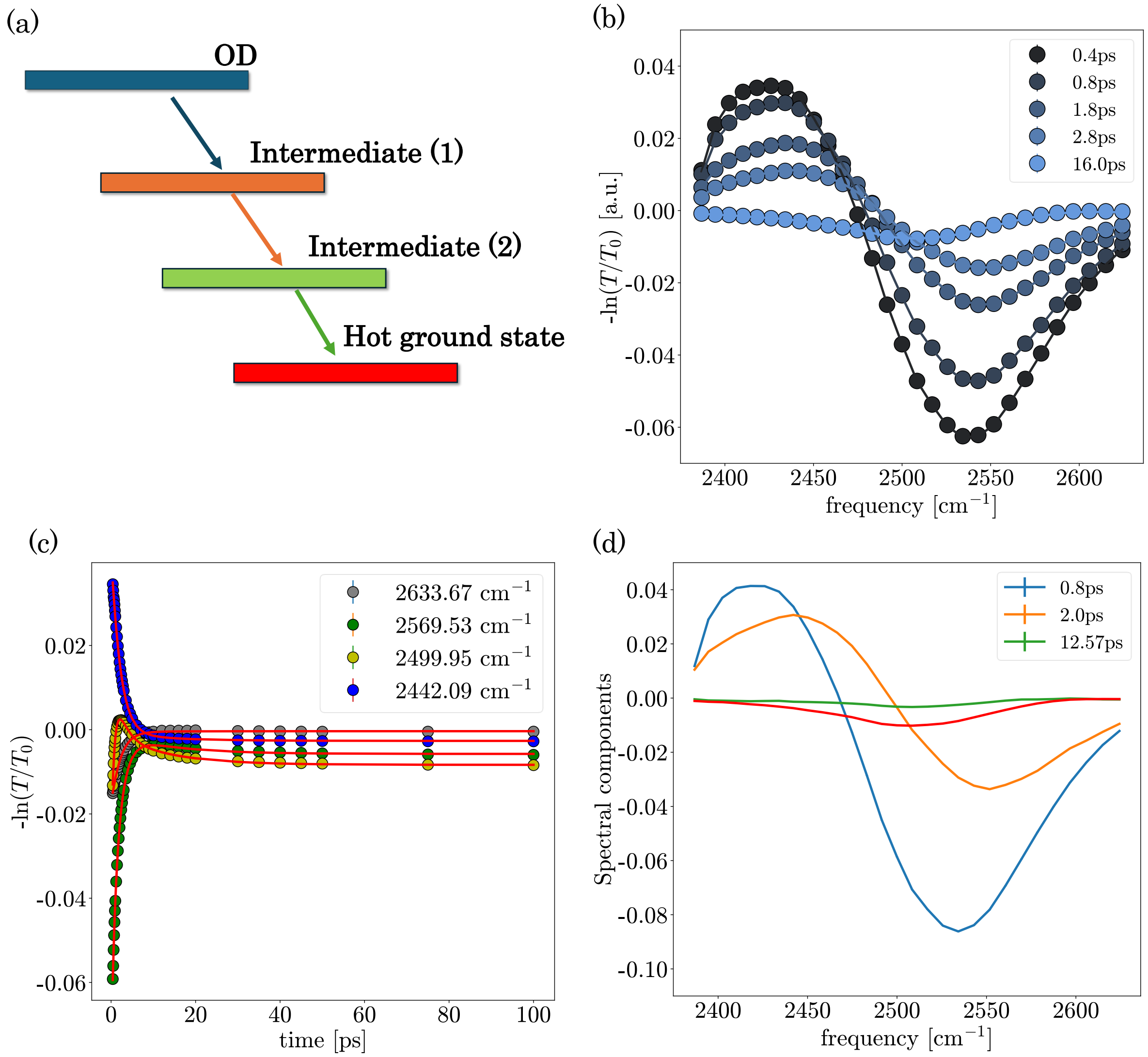

Figure S2: (a) Energy level diagram of the model that describes the vibrational relaxation of the OD stretch vibration of HFIP. (b) Isotropic absorption change as a function of frequency at different pump-probe delays. (c) Isotropic absorption change as a function of pump-probe delay at fixed frequencies. The curves in (b,c) show the result of the global-fitting procedure. (d) Spectral components of the vibrational levels extracted from the global fit. The red curve represents the hot ground state.

where k_1 and k_2, k_3 are the decay rates of each state. Each vibrational state is characterized by a transient absorption spectrum $\sigma_i(\omega)$, and the total time-dependent isotropic absorption change is

$$\Delta\alpha_{iso}(t,\omega) = \sum_{i=1}^{N} \sigma_i(\omega) N_i(t) \tag{S6}$$

where N is the total number of vibrational levels. The spectral components and the decay rates are finally obtained via a global least-squares fitting procedure on a weighted χ^2:

$$\chi^2 = \iint \left(\frac{\Delta\alpha_{iso}^{\text{experimental}}(t,\omega) - \Delta\alpha_{iso}^{\text{calc}}(t,\omega)}{\sigma(t,\omega)} \right)^2 d\omega dt \tag{S7}$$

Fig. S2-(b) and (c) show the isotropic absorption change for HFIP, along with the obtained fitting curve. An excellent agreement between the experimental data and the model can be clearly observed. Fig. S2-(d) shows the spectral components of each excited states along with the characteristic lifetime. Similar results were obtained for isopropanol, see Fig. S3.

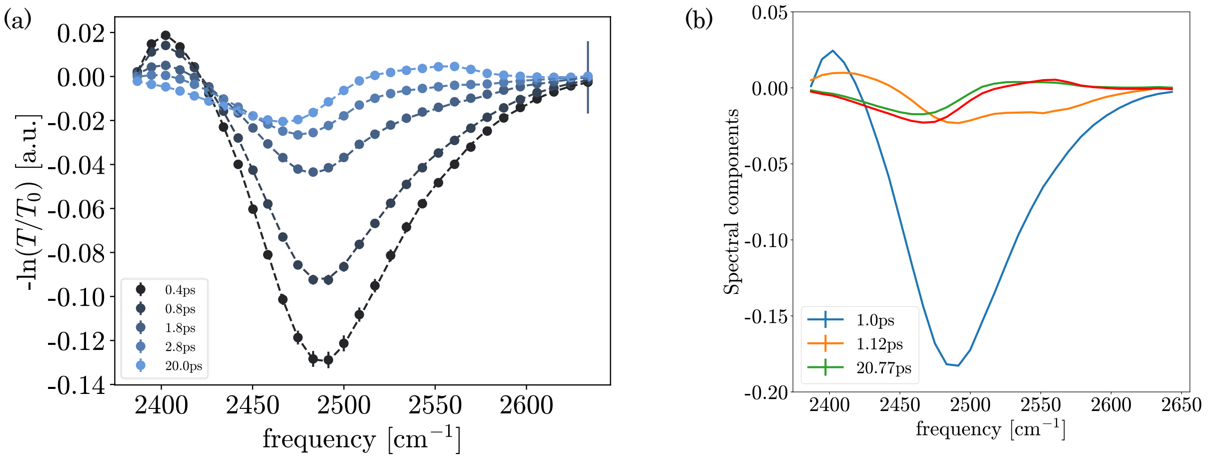

Figure S3: (a) Isotropic absorption change of the OD-stretch of isopropanol as a function of frequency at different pump-probe delays. The dashed lines are the curves obtained from the fitting procedure (b) Spectral components of the vibrational levels extracted from the fitting procedure. The red curve represents the hot ground state.

Table S2 lists the decay rates obtained from the global least-squares fits for HFIP and isopropanol. The excited-state decay rate $T_1 = 1/k_1$ is similar for the OD-vibration of HFIP and isopropanol and for the latter our value is in good agreement with the value of 0.990 ps

reported previously.[27]

Table S2: Time constants $T_i = 1/k_i$ associated to the vibrational dynamics of the OD-stretch mode of isopropanol and HFIP.

	Vibrational dynamics T_1 / ps	T_2 / ps	T_3 / ps
isopropanol	1.0 ± 0.20	1.12 ± 0.1	20.8 ± 0.2
HFIP	0.8 ± 0.2	2.0 ± 0.1	12.6 ± 0.1

Reorientation dynamics

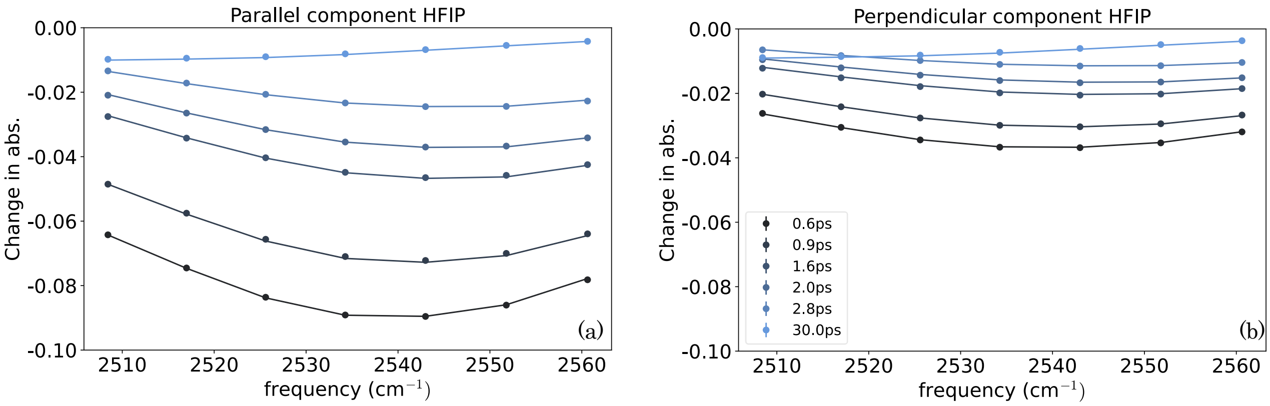

Figure S4: (a) Parallel ($\alpha_{||}$) and (b) perpendicular ($\alpha_{\perp}$) components of the absorption change measured in isotope diluted HFIP (D/H=0.1). The markers show the experimental data while the solid lines are the curves obtained by minimizing Eq. S11.

The analysis of the isotropic transient absorption can be used to analyze the reorientational dynamics and, in particular, to subtract the heating contribution from the $\Delta A_{||,\perp}$ signals. We use a procedure similar to that of refs. 25,26. The parallel and perpendicular components of the transient absorption signal can be expressed in terms of $R(t)$ (see Eq. S2), and using Eq. S6, we have

$$\Delta\alpha_{||} = \sum_{i}^{N}[1 + 2R(t)]N_i(t)\sigma_i(\omega) \tag{S8}$$

and

$$\Delta\alpha_{\perp} = \sum_{i}^{N}[1 - R(t)]N_i(t)\sigma_i(\omega) \tag{S9}$$

where $R(t)$ is the time-dependent OH-anisotropy, which we model as

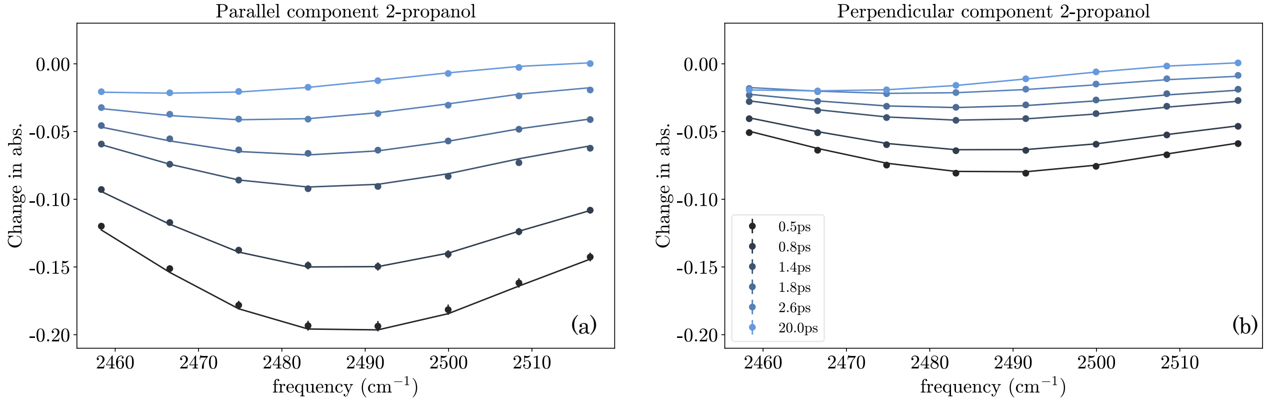

Figure S5: (a) Parallel ($\alpha_{||}$) and (b) perpendicular ($\alpha_{\perp}$) components of the absorption change measured in isotope diluted isopropanol (D/H=0.1). The markers show the experimental data while the solid lines are the curves obtained by minimizing Eq. S11.

$$R(t) = Ae^{-t/\tau_{\text{or}}}, \tag{S10}$$

where τ_{or} is the characteristic timescale of the OH-reorientation dynamics. A and τ_{or} are obtained by least-squares-fitting the parallel and perpendicular components via Eqs. S8 and S9 to the experimental data, specifically, by minimizing the function

$$\chi^2 = \int\int \left[\left(\frac{\delta\alpha_{||}(t,\omega) - \sum_i^N [1 + 2R_i]N_i(t)\sigma_i(\omega)}{\sigma^{||}(t,\omega)} \right)^2 + \left(\frac{\delta\alpha_{\perp}(t,\omega) - \sum_i^N [1 - R_i]N_i(t)\sigma_i(\omega)}{\sigma^{\perp}(t,\omega)} \right)^2 \right] d\omega dt\,. \tag{S11}$$

In the fitting procedure, both the spectral components $\sigma_i(\omega)$ and the population kinetics $N_i(t)$ are fixed to the values found from the analysis of the isotropic signal. The fits were performed in a frequency range centered at the bleach maximum, where the absorption change is largest. Examples of fitted parallel and perpendicular transient absorption signal are shown in Figures S4 and S5. Figure S6 shows the anisotropy that would be obtained

from the uncorrected $\Delta A_{||,\perp}$, together with a calculated reconstruction of this data using the parameters obtained from the above global least-squares fit. This shows that even without correcting for thermal effects, a similar difference in dynamics between HFIP and isopropanol is observed as in Figure 2 of the main text.

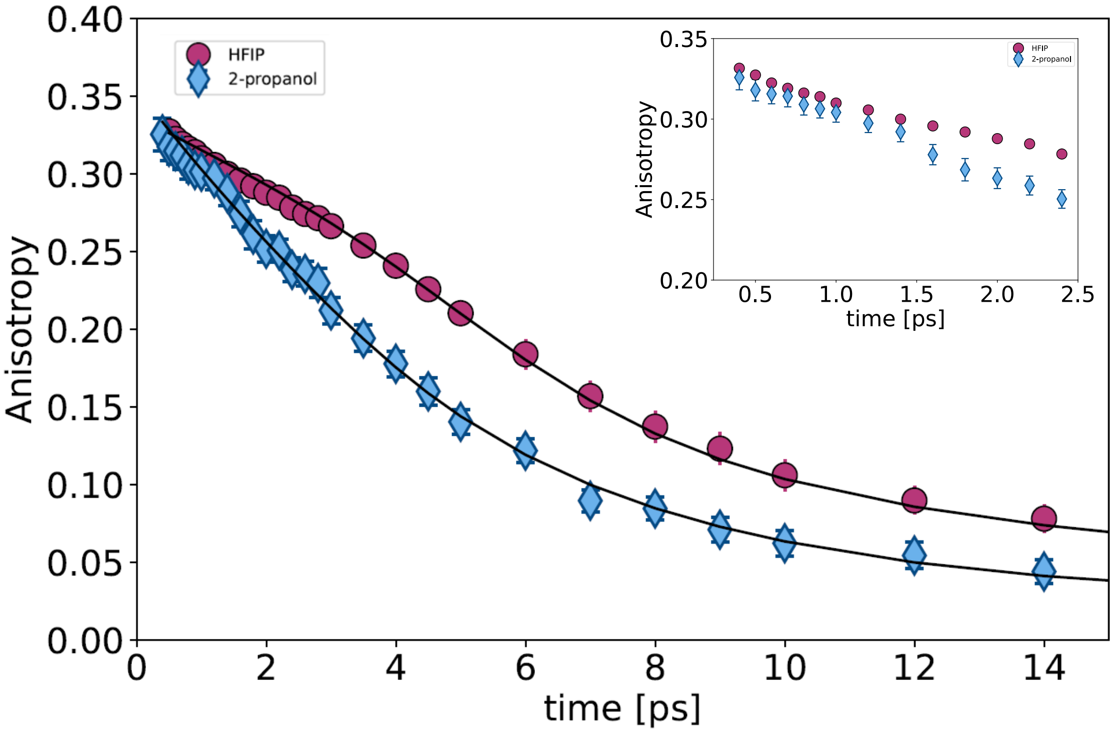

Figure S6: Total anisotropy as a function of delay time for HFIP and isopropanol isotope diluted solutions (D/H=0.1) at 2534 cm^{-1} and 2483 cm^{-1} respectively. The solid lines are fits to the experimental data via the model described in the supplementary information. The inset reports the first 2.5 ps of the anisotropy decay without further processing.

The time-dependent anisotropy of HFIP and isopropanol (the experimental data shown in Fig. 2 of the main text) were obtained by subtracting the thermal components obtained from the analysis described above from the experimental data:

$$\Delta\alpha_{||}^{corr}(t,\omega) = \Delta\alpha_{||}(t,\omega) - [(1 + 2R_{HG}(t))\sigma_{HG}(\omega)N_{HG}(t)] \quad \text{(S12)}$$

$$\Delta\alpha_{\perp}^{corr}(t,\omega) = \Delta\alpha_{\perp}(t,\omega) - [(1 - R_{HG}(t))\sigma_{HG}(\omega)N_{HG}(t)] \quad \text{(S13)}$$

where R_{HG}, $\sigma_{HG}(\omega)$ and $N_{HG}(t)$ are anisotropy, spectral component and population dynam-

ics of the hot ground state. Finally, the time-dependent anisotropy is obtained as

$$R(t) = \frac{\Delta\alpha_{||}^{corr} - \Delta\alpha_{\perp}^{corr}}{\Delta\alpha_{||}^{corr} + 2\Delta\alpha_{\perp}^{corr}}\,. \tag{S14}$$

Dynamics of isotopically diluted OH-groups

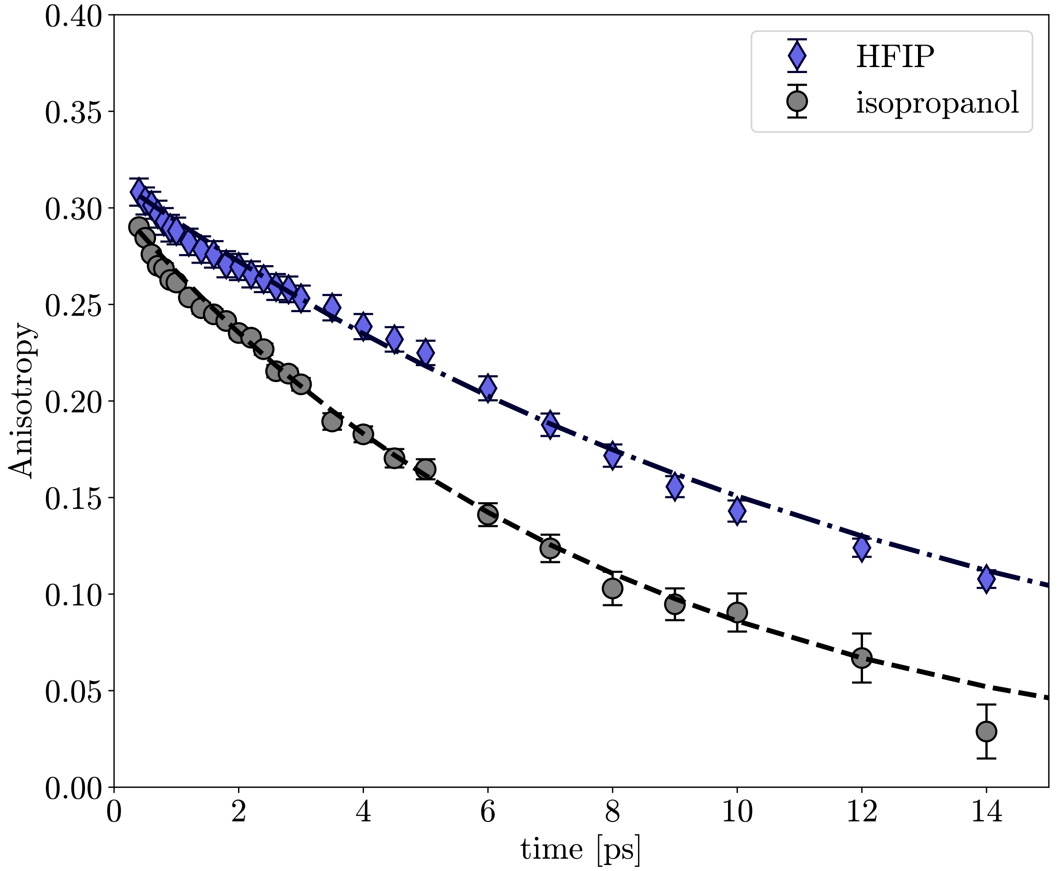

Figure S7: OH-Stretch anisotropy decay of hexafluoro-isopropanol (violet diamonds) and isopropanol (gray circles) measured at 3438 cm^{-1} and 3350 cm^{-1} respectively, i.e., at the center of the OH-stretch absorption band. The samples were isotopically diluted (H/D=0.1) to avoid coupling between the molecular oscillators. The solid lines show a least-squares fit of a single exponential decay to the data.

The reorientational dynamics of isopropanol and HFIP was also investigated in the inverted mix, that is, diluted HFIP-OH (isopropanol-OH) in HFIP-OD (isopropanol-OD). We used a dilution H/D=0.1. The anisotropy decays of the two molecules, extracted using the

procedure detailed in the previous sections, show an analogous trend as observed in Fig. 2 of the main text. The obtained τ^{or} are in quantitative agreement with the results obtained in D/H=0.1 solutions (see table below)

Table S3: Relaxation times τ_{or} obtained from least-squares fits to the time-resolved infrared data measured on H/D=0.1 isotope diluted solutions.

OH-stretch anisotropy	τ_{or}/ps
isopropanol	7 ± 1
HFIP	13.5 ± 1.6

Two-Dimensional Infrared Spectroscopy

Decay time maps

To explore frequency dependent vibrational relaxation dynamics in the alcohols we calculated decay time maps shown in Figure S9.[28] For this we integrated a 5x5 pixel grid for each pixel, and fit the decay of the integrated signal (from 250 fs to 2500 fs to exclude fast dynamics) with an monoexponential decay relaxing to a heated ground state[29] and display the decay times as a color map. We note that this approach neglects spectral diffusion dynamics, and spectral diffusion - as measured by the CLS dynamics (see main text) - will result in an apparent heterogeneity of the decay times. In this analysis we exclude pixels in the decay time maps that have less than 10% of the minimum signal at 250 fs waiting time as well as pixels with poor fit quality ($r^2 < 0.8$). Both HFIP and isopropanol show apparent frequency dependent vibrational lifetimes. Despite the slower spectral diffusion that should lead to less apparent lifetime heterogeneity (see Figure 3 of the main manuscript), HFIP shows more inhomogeneous decay times than isopropanol (standard deviation of the decay times for isopropanol: 300 fs and HFIP 400 fs). The more pronounced heterogeneity is also consistent with a higher amplitude of the intermediate state in figure S3. The more pronounced heterogeneity of decay times in HFIP than in isopropanol can also be explained by

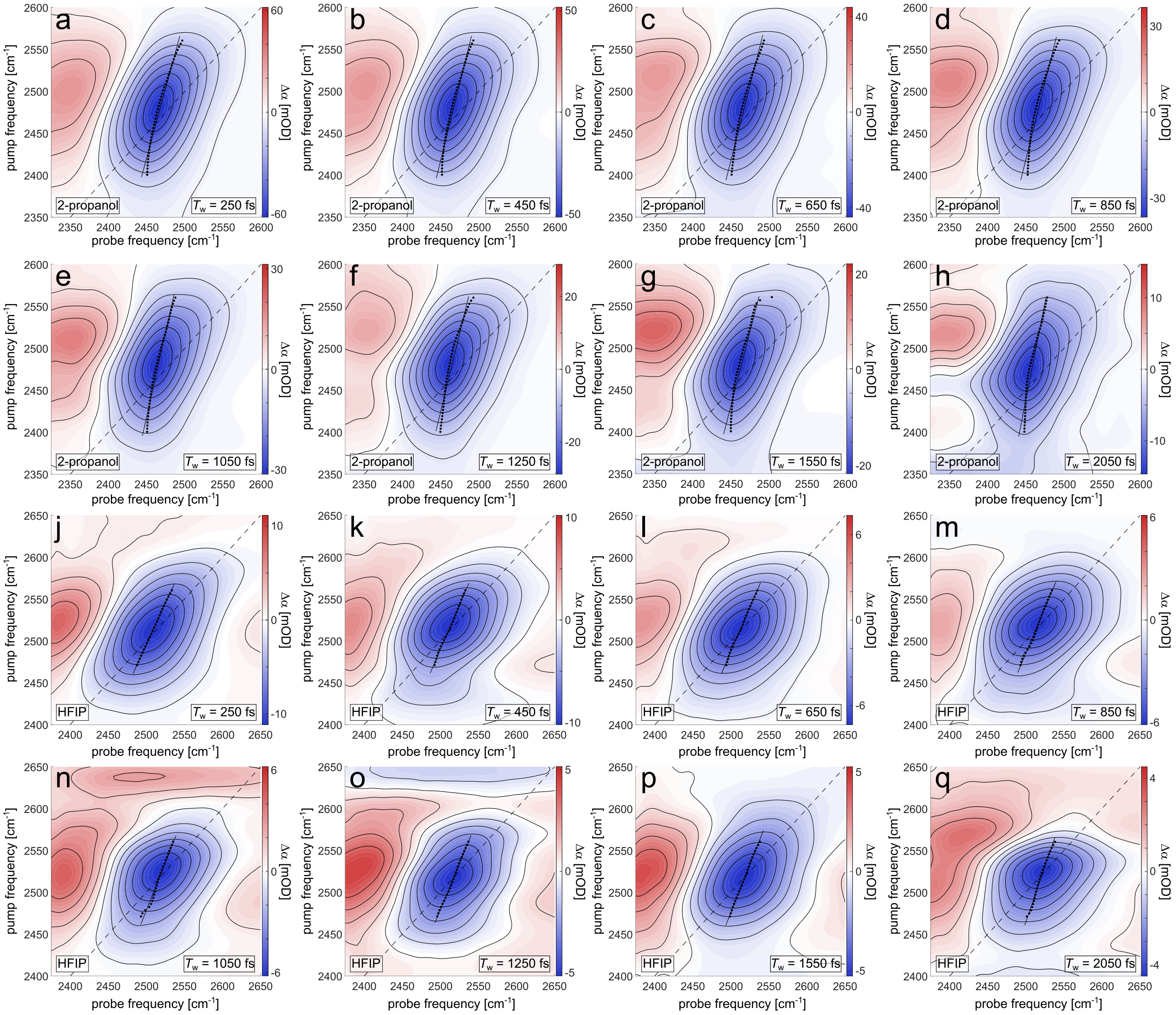

Figure S8: Additional 2D-IR spectra at different waiting times in $< ZZZZ >$ polarization (parallel) of a-h) isopropanol and j-o) HFIP with an isotope ratio D:H of 1:9. The minima parallel to the probe axis (center line points) are indicated with black symbols and the center line is displayed as solid line.

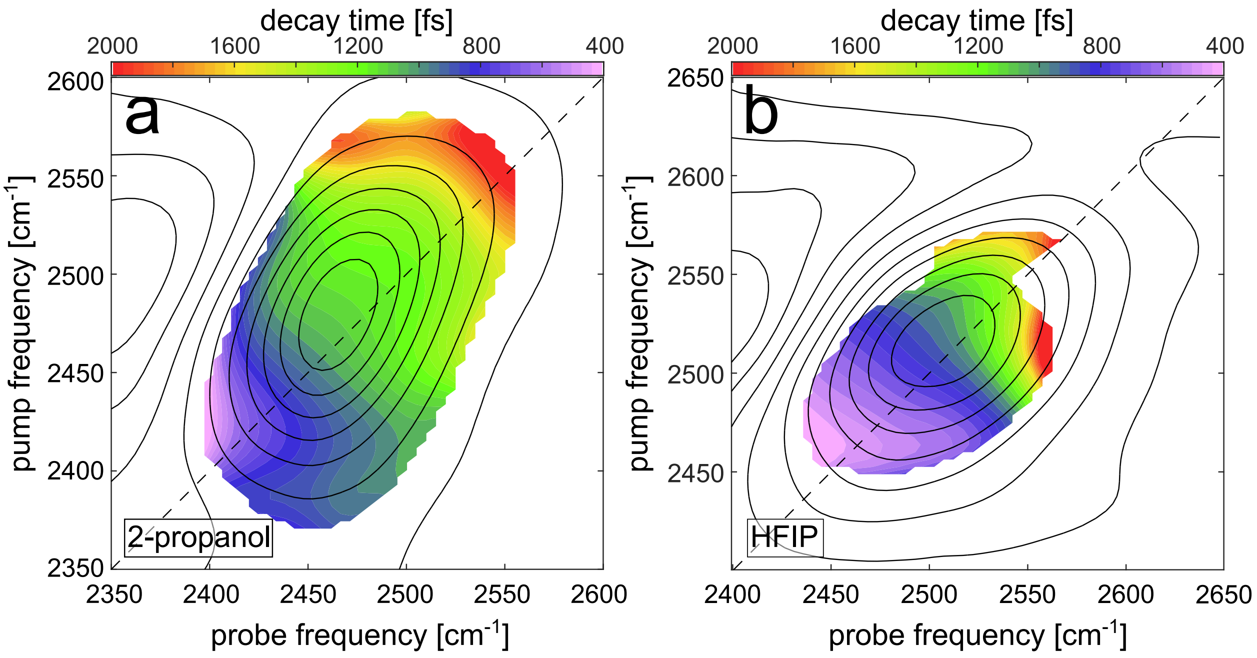

Figure S9: 2D-IR spectra at T_w = 100 fs in $< ZZZZ >$ polarization (parallel) of a) isopropanol and b) HFIP with an isotope ratio D:H of 1:9 (contour plot). Decay time maps are displayed as a color map.

a larger contribution of terminal OD groups of hydrogen-bonded HFIP chains than for isopropanol chains: Non-bonded, terminal OD groups (β) have a blue-shifted OD(OH) stretch frequency and do therefore not contribute to the main hydrogen-bonded IR bands (see SI Fig S1), however hydrogen-bonded molecules at the end (γ) of hydrogen-bonded chains are spectrally less shifted than OD groups in the middle (δ) of the chains as well as branching molecules (ϵ).[27,30] These different subspecies therefore contribute to inhomogeneous broadening of the main, hydrogen-bonded OD peak. The ratio of these subspecies is linked to the size of the aggregates: longer chains are dominated by δ, while short chains have significant contributions from γ and more branched chains from ϵ and γ motifs. The vibrational lifetimes of these different species have been suggested to differ.[27,30,31] As such, different hydrogen-bonded chain lengths will results in differing heterogeneities in the signal decay times: The more heterogeneous decay times for HFIP support the notion that HFIP forms smaller clusters than isopropanol.

Hydrogen-bonding energies from DFT calculations

To computationally asses the differences in hydrogen-bond donor and acceptor strengths, we calculated the interaction energies of hydrogen-bonded dimers using DFT. Note that these energies are solely based on the electronic single point energies (see below for Cartesian coordinates and single point energies) and are not corrected for zero point energies, vibrational, or entropic terms. As such, these calculations merely serve for qualitative comparison of trends in the hydrogen-bond donor and acceptor abilities of the alcohols. As apparent from these calculations in Figure S10, the hydrogen-bonding energies between two isopropanol and two HFIP molecules are comparable. These similar interaction energies are, however, a result of HFIP being a poorer hydrogen-bond donor (reduced interaction energy for isopropanol donating a hydrogen-bond to HFIP) and the enhanced hydrogen-bond donor strength (enhanced interaction energy of HFIP donating a hydrogen-bond to isopropanol) relative to isopropanol-isopropanol hydrogen-bonding. The enhanced hydrogen-bond donor strength of HFIP relative to isopropanol is further illustrated by the more favorable interaction with diethylether (Figure S10).

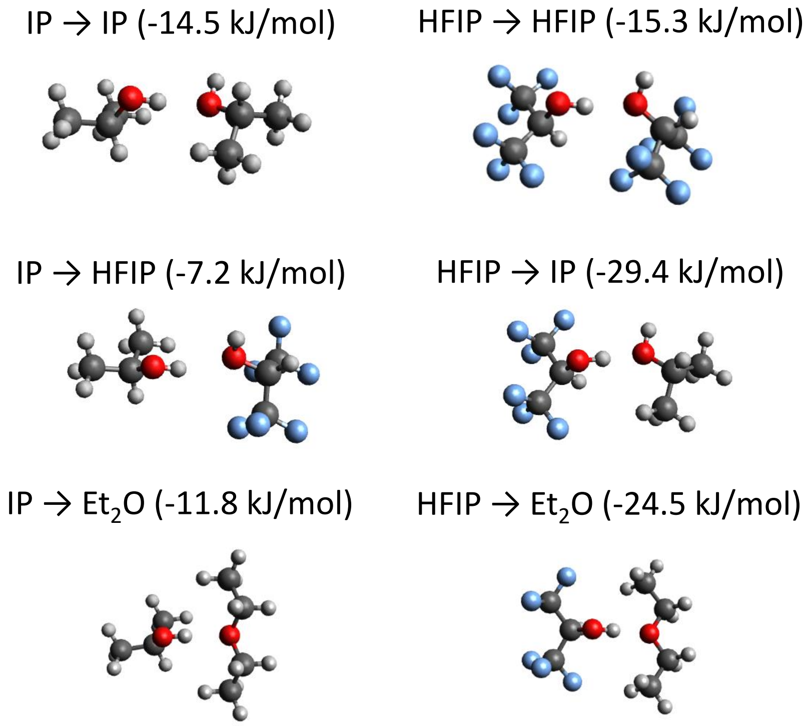

Figure S10: DFT-optimized geometries of hydrogen-bonded dimers of isopropanol (IP) and hexafluoroisopropanol (HFIP). Top row: homo-dimers; center row: hetero-dimers; bottom row: alcohol-diethylether dimers. Arrows indicate hydrogen-bond donation. Energies in parentheses refer to interaction energies as obtained from the DFT calculations (see text).

Association constants obtained from linear IR spectra

Figure 1 in the main manuscript shows linear absorption spectra of HFIP (blue) and isopropanol (red) at 1, 2, 5, and 10 mol% in chloroform. For HFIP we observe a peak at 3600 cm^{-1}, which corresponds to the free OH peak. At higher HFIP concentrations a red shifted shoulder appears at about 3400 cm^{-1}, that is likely caused by self association of HFIP molecules. For low concentrations of isopropanol the spectrum is also dominated by the free OH peak at about 3615 cm^{-1} but with increasing concentration a bonded peak at about 3400 cm^{-1} grows in. Together the spectra show that self association is much more favourable for isopropanol compared to HFIP, where even at 10 mol% the free form still dominates. This can be explained with HFIP being a much weaker hydrogen bond acceptor.

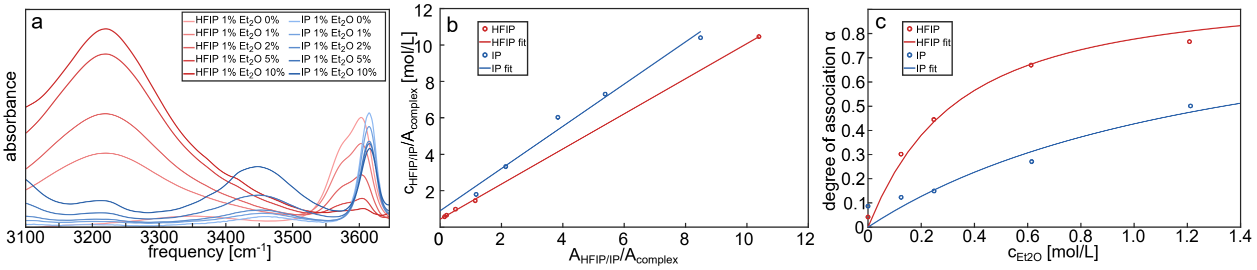

Figure S11: a) IR absorption spectra of 1 mol% HFIP (red)/isopropanol (blue) (IP) in chloroform with increasing amounts of diethylether as hydrogen-bond acceptor. Data show spectra with a scaled solvent background subtracted. b) Plot of $\frac{c^0_{\text{HFIP/IP}}}{A_{\text{complex}}}$ vs $\frac{A_{\text{HFIP/IP}}}{A_{\text{complex}}}$ with a linear regression according to equation S19 to obtain extinction coefficients. c) Degree of association as a function of diethylether concentration. Symbols show data extracted from spectral amplitudes and solid lines show fits with the bimolecular association model (Equation S15).

To quantify the hydrogen-bonding of the alcohols to a representative interaction motif of a reactant we measured linear absorption spectra of HFIP and isopropanol with increasing concentrations of diehylether dissolved in chloroform (see Figure S11). From these spectra we calculate the association constants for alcohol-diethylether association using a bimolecular association model (Equation S15).[32]

$$K_{\text{ass}} = \frac{[complex]}{[HFIP/IP][Et_2O]} \tag{S15}$$

with the association constant K_{ass}, the equilibrium concentration of the alcohol-diethylether complex $[complex]$ and the equilibrium concentrations of the free alcohol, $[HFIP/IP]$, and free diethylether $[Et_2O]$. To relate the measured absorption A (average absorption for HFIP bonded: 3100-3500 cm^{-1}, HFIP free: 3520-3630 cm^{-1}, IP bonded: 3300-3570 cm^{-1} and IP free: 3570-3640 cm^{-1}) to the concentrations of free and complexed forms we use Beer-Lamberts law (see Equation S16 and S17).

$$A_{\text{HFIP/IP}} = \epsilon_{\text{HFIP/IP}} \cdot [HFIP/IP] \cdot d \tag{S16}$$

$$A_{\text{complex}} = \epsilon_{\text{complex}} \cdot [complex] \cdot d \tag{S17}$$

with the sample thickness $d = 200\ \mu$m. To obtain the extinction coefficients $\epsilon_{\mathrm{HFIP/IP}}$ and $\epsilon_{\mathrm{complex}}$ we substitute Equation S16 and S17 into the relation for the total alcohol concentration $c^0_{\mathrm{HFIP/IP}}$ Equation S18. To determine the total alcohol concentration we assume ideal mixing (volumes).

$$c^0_{\mathrm{HFIP/IP}} = [HFIP/IP] + [complex] \tag{S18}$$

yielding Equation S19:

$$\frac{c^0_{\mathrm{HFIP/IP}}}{A_{\mathrm{complex}}} = \frac{1}{\epsilon_{\mathrm{HFIP/IP}} \cdot d} \cdot \frac{A_{\mathrm{HFIP/IP}}}{A_{\mathrm{complex}}} + \frac{1}{\epsilon_{\mathrm{complex}} \cdot d} \tag{S19}$$

From linear regression of $\frac{c^0_{\mathrm{HFIP/IP}}}{A_{\mathrm{complex}}}$ vs $\frac{A_{\mathrm{HFIP/IP}}}{A_{\mathrm{complex}}}$ (Figure S11 b) we obtain the extinction coefficients from the slope and the intercept. Using these extinction coefficients we determine the equilibrium concentrations of free and complex species at each concentration.

To finally obtain the association constants K_{ass} we plot the degree of association α (see Equation S20) vs concentration of diethylether $c^0_{Et_2O}$

$$\alpha = \frac{[complex]}{c^0_{HFIP/IP}} \tag{S20}$$

and fit

$$\alpha = \frac{1}{2 \cdot K_{\mathrm{ass}} \cdot c^0_{\mathrm{HFIP/IP}}} \Bigg(K_{\mathrm{ass}} \cdot c^0_{\mathrm{HFIP/IP}} + K_{\mathrm{ass}} \cdot c^0_{\mathrm{Et_2O}} + 1 - \sqrt{K^2_{\mathrm{ass}} \cdot {c^0_{\mathrm{HFIP/IP}}}^2 - 2K^2_{\mathrm{ass}} \cdot c^0_{\mathrm{HFIP/IP}} \cdot c^0_{\mathrm{Et_2O}} + 2K_{\mathrm{ass}} \cdot c^0_{\mathrm{HFIP/IP}} + K^2_{\mathrm{ass}} \cdot {c^0_{\mathrm{Et_2O}}}^2 + 2K_{\mathrm{ass}} \cdot c^0_{\mathrm{Et_2O}} + 1} \Bigg) \tag{S21}$$

which is based on Equation S15 and S20 and mass conservation (Figure S11 c). Form these fits we obtain the association constants $K_{\mathrm{ass}}(\mathrm{IP}) = 0.8\ \frac{\mathrm{L}}{\mathrm{mol}}$ and $K_{\mathrm{ass}}(\mathrm{HFIP}) = 3.9\ \frac{\mathrm{L}}{\mathrm{mol}}$.

Optimized Cartesian coordinates obtained from DFT calculations

IP-IP dimer

IP-IP

FINAL SINGLE POINT ENERGY -388.619213507818

C	-0.69936655532737	1.07606519496207	-0.09622805530488
C	0.78489238171738	0.78701168794454	0.08863420740594
O	-1.17523930493686	1.64926212534575	1.13277625415041
C	-1.49584653205521	-0.17617820716148	-0.46562742101847
H	-0.81645985887085	1.81749659548525	-0.89997331556071
H	0.93331541099217	0.05916807360746	0.89294664251117
H	1.32607145813287	1.70118978945119	0.34664669194275
H	1.21496765119488	0.37860536811464	-0.83005720698014
H	-1.38941247255697	-0.93876832040834	0.31266098161560
H	-1.14046575081378	-0.59492155215849	-1.41254600109740
H	-2.55871917584989	0.05740624042420	-0.57784475242920
H	-2.09959694170397	1.93541205123738	1.01310227694594
O	-3.89858205355720	2.48066447641060	0.91871533713185
C	-4.31674114241165	3.85084108447949	1.15181847999359
H	-4.06964197452041	4.10940144594221	2.18941444390774
C	-5.82118839086317	3.99094319468466	0.94360189222126
C	-3.50140891262995	4.72443638936233	0.21057195296744
H	-4.43240645881561	1.89763218462825	1.47292799451798
H	-2.43108526313231	4.58263320480761	0.37917284726004
H	-3.73832466946646	5.77851512493960	0.37665226104833
H	-3.72695117020319	4.47899958186183	-0.83173899162535

H	-6.37321795739653	3.32777166183926	1.61735829909176
H	-6.09184431721580	3.74358451443778	-0.08738496173769
H	-6.13922799970997	5.01711808976205	1.14985014304201

IP-IP constrained

FINAL SINGLE POINT ENERGY -388.613688137588

C	23.83564628933190	-10.60939752523594	-1.94286903133818
C	25.24203896463911	-11.18022184729525	-1.83109662529407
O	23.48054199700651	-10.12321417956266	-0.63018392250014
C	22.81808815915103	-11.63422355679222	-2.43893150782209
H	23.85061307224656	-9.75741372991140	-2.63656425996759
H	25.25686695623476	-12.02584744031881	-1.13635990665671
H	25.93916328083676	-10.42072881853356	-1.46798253692323
H	25.58970476082298	-11.52857389035100	-2.80730230745933
H	22.79020451173745	-12.50147205111321	-1.77186669429012
H	23.07786779858375	-11.97739303263983	-3.44497505242807
H	21.81480723074515	-11.19804162169246	-2.48210582075359
H	22.58733729363151	-9.76168574600163	-0.67124943481967
O	-3.68667892146827	2.53241767247209	0.70289070928730
C	-4.19773555119415	3.83115369196195	1.07383947174472
H	-3.79945422932807	4.09109211249886	2.06433291703520
C	-5.72320358186750	3.81750414319126	1.14049981242012
C	-3.65941348807919	4.81983923961759	0.04964694935222
H	-4.00553572852136	1.88628991360863	1.34401435379321
H	-2.56683360699579	4.79698607256706	0.02877279022294
H	-3.97815821848831	5.83586381409042	0.29720094489766
H	-4.03147761240843	4.57426424320252	-0.94992661123641
H	-6.07497711131860	3.07786269888089	1.86701024792149

H	-6.14742511335334	3.57091885645821	0.16223775906279
H	-6.10318715194576	4.79657098089891	1.44773775575159

HFIP-HFIP dimer

HFIP-HFIP

FINAL SINGLE POINT ENERGY -1579.518435370187

C	-1.21977430062144	0.87168568339964	-0.22449485363802
C	0.21322632316142	1.34570146632308	-0.53677461966691
O	-1.47756987859573	1.10095252263753	1.12978733672660
C	-1.45768951455938	-0.62049890477287	-0.53167198989732
H	-1.88308912820445	1.43664131506583	-0.88642889969136
F	1.14204554514102	0.70192177451243	0.19393727087255
F	0.32291729571062	2.65964143805045	-0.26009095706923
F	0.52714518085489	1.17997589229054	-1.83761042149995
F	-0.69665315715443	-1.43255996153550	0.22463606987772
F	-1.22089002185204	-0.92077918405583	-1.82385614163889
F	-2.74722082921350	-0.92505563844992	-0.27770328512917
H	-2.27106381154545	1.65562652340256	1.22442268203473
O	-3.85517071009938	2.58174504684350	1.63832589576068
C	-4.28483964911913	3.91066157932941	1.45667067389144
H	-4.69780496994620	4.33894261390585	2.37405290543060
C	-5.41256246846787	3.93771708858067	0.40356842095816
C	-3.06074855183896	4.76741784709334	1.08072563119452
H	-4.22220935986964	2.22673460885959	2.45859156278688
F	-2.13174336773114	4.67115014565200	2.04853938698181
F	-3.39026267920076	6.06525973454850	0.95355235593431
F	-2.49036940800757	4.37224536497365	-0.07211005209974

F	-6.43343289555767	3.17113677024478	0.83454157314540
F	-5.02149959931389	3.45572797304961	-0.78882289147427
F	-5.88722004396671	5.18229830004880	0.21366234620903

HFIP-HFIP constrained

FINAL SINGLE POINT ENERGY -1579.512592590798

C	16.66041009690571	-19.20335815874020	0.73045061354281
C	16.66027872560048	-20.58884657361490	1.40818495800954
O	16.33566054818867	-19.36445071392096	-0.62409466154296
C	18.01044095590059	-18.46436694478075	0.82352349086348
H	15.93285587017255	-18.59329552236888	1.27364163374089
F	17.54595337116926	-21.43828018781074	0.85817806394806
F	15.43768428234048	-21.14264232802039	1.28541930030836
F	16.93505823004602	-20.50386978034437	2.72439585182861
F	18.99792037810939	-19.10704128270426	0.17565168276430
F	18.40152407272618	-18.28709895174277	2.10071859811523
F	17.88803958975570	-17.24369399020625	0.26462207426217
H	15.51358921612790	-18.89867317632465	-0.81793338694576
O	-3.28265382108453	3.33074035943092	-0.40039175122118
C	-3.79425635788471	3.97764497720586	0.73273749649698
H	-3.69920877253608	5.06662610457327	0.68172241023811
C	-2.95440447135381	3.52196046211452	1.93983530229162
C	-5.30242537346299	3.67280598914801	0.86678540470443
H	-3.22780961276004	3.95619925311973	-1.13289457762644
F	-5.93040178376661	4.07204779590755	-0.25927710573135
F	-5.86385718813287	4.33189141707142	1.89861874525800
F	-5.55860834532717	2.36299590348052	1.02709122574296
F	-1.67834356139069	3.92349518661698	1.77595852266438

F	-2.94120783147716	2.18548956111795	2.08463635780277
F	-3.40324821787847	4.05672060080689	3.09149975048330

IP-HFIP dimer

IP-HFIP

FINAL SINGLE POINT ENERGY -984.065846174991

C	-0.62312235970652	0.93413311682941	-0.27364869632303
C	0.72737444781040	0.26613123131461	-0.05488346468402
O	-0.91593619136553	1.68379426831313	0.92230562160235
C	-1.73392955223948	-0.07016725313557	-0.57666592512935
H	-0.54048369915374	1.63660237683560	-1.11470262451543
H	0.67367704561888	-0.43792734279000	0.78172734682515
H	1.49510577611466	1.01143365641079	0.16856371214305
H	1.03111961366623	-0.28406290912315	-0.94956083423422
H	-1.83148634849827	-0.79090016347149	0.24139921428507
H	-1.51558602710336	-0.61865434928172	-1.49822255521262
H	-2.69433334751510	0.43802955956664	-0.70741307432043
H	-1.76342299808901	2.13655676153342	0.81162707152753
O	-3.55130029193381	2.98446215537848	1.56004571000181
C	-4.44783959685349	4.04557482545216	1.35234686316433
H	-4.87925717313574	4.42029775784734	2.28461409745497
C	-5.62265168687856	3.53813928412480	0.49251445488176
C	-3.67096107919406	5.21984914263688	0.72420905036992
H	-3.54228610061780	2.74009066525194	2.49364334858467
F	-2.73197843396281	5.64483826043509	1.59204255546824
F	-4.47203205224778	6.26639377765242	0.45061774724610
F	-3.04559698639423	4.87145486647137	-0.41433524160899

F	-6.24039001347196	2.52796726106479	1.13705914367982
F	-5.22195064951639	3.07014067103126	-0.70315273720056
F	-6.53921229533193	4.50011237965123	0.27531921599378

IP-HFIP constrained

FINAL SINGLE POINT ENERGY -984.063109080579

C	18.52016103599816	-18.05807429722210	-4.32553648231809
C	19.66996485015529	-18.08643160297887	-3.32908840835925
O	17.56885016367045	-17.09011244121816	-3.83178232645019
C	17.86299054262343	-19.42412162803238	-4.51059845383638
H	18.89832577579174	-17.70974913526479	-5.29659337749148
H	19.31795183073667	-18.42315049813787	-2.34897018503560
H	20.10797625980878	-17.09129367680191	-3.21736732598181
H	20.45150328222338	-18.77078538256839	-3.66978982324911
H	17.48387532430415	-19.79992479605318	-3.55517580906032
H	18.58349971162621	-20.14596255338650	-4.90706329835559
H	17.02718053443264	-19.36285735936337	-5.21509241023411
H	16.82786657688522	-17.05035828238127	-4.44790750669768
O	-3.14858415552753	3.86429709795134	1.79854926867189
C	-4.45173303456334	4.20984477091422	1.41535784812217
H	-5.14678819829733	4.24896162052245	2.25921063840686
C	-4.99841378449443	3.14377113993957	0.44138412504636
C	-4.40479479782814	5.63015192488101	0.81845283210095
H	-3.15450966961653	3.52824617351991	2.70262935929274
F	-4.01747184150117	6.50475280723089	1.76749288890614
F	-5.61267574204974	6.02617535347456	0.37189346784774
F	-3.53556474318125	5.73356816300788	-0.20141217358070
F	-4.95377140601662	1.93366907037810	1.03534686029074

F	-4.28211582502623	3.06240812613931	-0.69404522910445
F	-6.28095269016048	3.37939540545522	0.10423552107085

HFIP-IP dimer

HFIP-IP

FINAL SINGLE POINT ENERGY -984.074369320203

C	-1.09065565238304	0.87947977561398	-0.05793176721708
C	0.36863479506192	1.32056531586787	-0.27963943023953
O	-1.48638740906151	1.29316616676160	1.21131067489145
C	-1.29708388801232	-0.64410186314144	-0.20413703365644
H	-1.67609749215834	1.34429515120168	-0.85883917051827
F	1.20412950192653	0.85182653222878	0.66532554778780
F	0.44069522118517	2.66702249432425	-0.24774066792434
F	0.84422038333189	0.92125635795811	-1.47764847171889
F	-0.53398500224393	-1.36381530261830	0.63996024826185
F	-1.03705519928097	-1.07540151702637	-1.45583500718414
F	-2.58486891806907	-0.94507621227480	0.06206794869141
H	-2.34809677643249	1.77076320158340	1.14860012076108
O	-3.89423298105449	2.48147446705118	0.96739319854648
C	-4.27913500815622	3.86470451865496	1.21359357377365
H	-4.35689357610091	4.00139121671844	2.29879497067357
C	-5.62911155065311	4.15246858416655	0.56669079663063
C	-3.15859146297690	4.73876314256217	0.67383198879833
H	-4.59053753720089	1.89837275858283	1.29582402401530
H	-2.20809770743792	4.49988183505545	1.15707397901250
H	-3.38392433824501	5.79106904154257	0.86454595468366
H	-3.04865295384973	4.59993906779774	-0.40592872291847

H	-6.40092813767017	3.48046280169932	0.95488262734930
H	-5.57029504544069	4.02679908091357	-0.51851853182653
H	-5.93952926507743	5.17898338477619	0.78177314932659

HFIP-IP constrained

FINAL SINGLE POINT ENERGY -984.063156029618

C	15.84347739610987	-19.86125446468805	-5.33506584577116
C	15.65401198893107	-20.11733749574924	-3.82443229989959
O	14.88449837330096	-20.58811047384026	-6.05407215571630
C	17.23170646745697	-20.26412794203553	-5.86704632365574
H	15.75658880888548	-18.78068318856447	-5.48172171150560
F	15.79080923101437	-21.41430716142465	-3.49680526001818
F	14.40969233035113	-19.73812527708710	-3.46793108832471
F	16.51814118410084	-19.40766316666872	-3.07410196471584
F	17.45591637956782	-21.58661624206630	-5.77828691364702
F	18.22158807955228	-19.63250914776136	-5.20614996794285
F	17.33137732541031	-19.92387857462147	-7.16683201373114
H	14.17202367053256	-19.99929230037722	-6.33085119922671
O	-3.56597530439434	2.08057673670079	0.70689214985591
C	-3.83976254914257	3.48938561488102	0.54300578404576
H	-2.95756666013221	3.96415999760546	0.09193440168843
C	-4.12493043381635	4.15065228310686	1.88954777002259
C	-5.00873946840545	3.59799730755885	-0.42538249333270
H	-2.81999454901678	1.98436306415931	1.31069701832332
H	-4.77176372241929	3.10864296903098	-1.37356480626967
H	-5.23867012569068	4.64758930958638	-0.62726600495812
H	-5.90001096293711	3.12378457067088	-0.00264879591203
H	-3.27642283537550	4.03125064091635	2.57096710096078

H	-5.00896932177658	3.70501337057812	2.35621751252948
H	-4.30305530211751	5.22259957010356	1.76056710720519

IP-Et_2O dimer

IP-Et2O

FINAL SINGLE POINT ENERGY -427.898517826805

C	-0.89284384738190	0.91701909682353	-0.14084997555989
C	0.60699094407371	1.03788059606186	-0.37919430598540
O	-1.23452845501866	1.86258813365892	0.88723811788765
C	-1.30764593267027	-0.50127739546973	0.25266728134850
H	-1.42037897357052	1.19522040863256	-1.06444904101852
H	1.15858443499814	0.78119601158644	0.53102902279031
H	0.87098593276020	2.05908319286273	-0.66636809346347
H	0.92540128668503	0.36144579078092	-1.17716604147839
H	-0.78481684855538	-0.81252955106784	1.16268796050561
H	-1.06477125896752	-1.20923913006467	-0.54608198791442
H	-2.38443456555382	-0.55591069515311	0.43703971285909
H	-2.20356600835876	1.90184478983886	0.97841617153218
H	-2.95727162151388	0.85876693788540	3.26040244965723
H	-3.10169193642577	3.25254675313704	-1.03327382041750
H	-4.09637706208062	3.10014089024825	3.13919147660379
H	-4.16399326339993	4.29681512762909	0.99514809204526
C	-4.04694780037980	0.93724457992327	3.27928133020931
C	-4.51434833311869	2.22401271328307	2.62451198816309
O	-4.09939180467994	2.23956078165782	1.25182306850187
C	-4.18818512879950	3.28130714209659	-0.92000372565741
C	-4.58271428462955	3.38790031771947	0.54157614821638

H	-4.37308957396725	0.91679850273561	4.32296377845838
H	-4.56514996579240	4.14993861972080	-1.46715773595533
H	-4.46889525504496	0.06619862194189	2.77100769178921
H	-4.61154295234536	2.38086583082704	-1.37300577576351
H	-5.60904829512250	2.30295558066901	2.66395339991921
H	-5.67504943113970	3.43424635203493	0.64505281272668

IP-Et2O constrained

FINAL SINGLE POINT ENERGY -427.894019901879

C	22.23852150619493	-9.82217359611176	-9.69485227720503
C	23.75545322880639	-9.90099492543079	-9.78936329131254
O	21.93883284415675	-8.90610819253626	-8.61931888690257
C	21.58868641059225	-11.18198049260186	-9.44964644525703
H	21.84405361431071	-9.40116554486828	-10.62993280754424
H	24.17484404092586	-10.30750825490676	-8.86375930775382
H	24.18355855242675	-8.90955407251162	-9.95804656220332
H	24.05218979123300	-10.54981508370800	-10.61770951514230
H	21.97124062091125	-11.62826858544183	-8.52646151582136
H	21.79993322239795	-11.86346480068176	-10.27925922281995
H	20.50156019680618	-11.08539532162254	-9.36324707649284
H	20.98043059353737	-8.83622015859324	-8.53742395911800
H	-2.80023274921429	0.87232866469991	3.11078475079322
H	-3.18353119966985	3.06980595902708	-1.10165796548423
H	-3.92247873706430	3.12003496921269	3.03919231736734
H	-4.11559458815354	4.22306158070415	0.92753309655387
C	-3.87826768844083	0.96653512461688	3.26702196570221
C	-4.40885461856761	2.23000736277459	2.61358408656072
O	-4.15031303487748	2.17122131547458	1.21171551239207

C	-4.26284316725546	3.17514475824749	-0.96242752295291
C	-4.60091337774186	3.32877976424257	0.50971237694625
H	-4.06799677832664	0.99396831014359	4.34384675010344
H	-4.59740081052715	4.05570229830867	-1.51815774930267
H	-4.36799872273303	0.08019514616143	2.85478131835162
H	-4.75509384261100	2.29486939924267	-1.38451528545109
H	-5.49089206334030	2.33049831798668	2.78378670367568
H	-5.68573324377635	3.44456605817175	0.64984051231763

HFIP-Et_2O dimer

HFIP-Et2O

FINAL SINGLE POINT ENERGY -1023.352851163025

C	-1.47642211292352	1.02125594229483	0.19465006554473
C	0.03345435898455	1.31385655630836	0.34339012404000
O	-2.09124221246915	1.15538347417714	1.43663124828372
C	-1.77105939625690	-0.38708283202104	-0.36405856267769
H	-1.85614051852696	1.72820804092600	-0.55070845270160
F	0.59879511320928	0.61408919709600	1.34281592305322
F	0.20746498177244	2.62372053909168	0.62408270215229
F	0.72818177310840	1.05170826261847	-0.78273100504963
F	-1.23243280517754	-1.37202619145145	0.38061512481911
F	-1.31190494138992	-0.53407036646079	-1.62473917437778
F	-3.10213265064943	-0.59112575413181	-0.39826784718479
H	-2.67043420886327	1.95172346112169	1.45543086785331
H	-1.22927141976710	4.35761728379878	2.08771686337866
H	-4.71745549363389	2.14072232020190	-0.54902268895811
H	-3.50809526951754	5.40264287416689	1.88650353066819

H	-5.20389850192595	4.33227880238687	0.59375115837860
C	-1.92213719420274	4.36479052642243	2.93188913026648
C	-3.35948980084847	4.48045127318052	2.46304080566964
O	-3.70047939322597	3.35934532053815	1.62849356062472
C	-5.37076843275490	2.18994265861458	0.32609027581570
C	-5.06260651091635	3.40993440065518	1.17198193080793
H	-1.68020307927864	5.22347355173158	3.56441052740530
H	-6.40492100537913	2.24438427965285	-0.02493245231767
H	-1.76914961196024	3.45591080758584	3.51914871958577
H	-5.25485566149292	1.26929060830855	0.90307566753305
H	-4.04742596853197	4.50292137264816	3.31807298715797
H	-5.72529003738183	3.45517359053839	2.04589897022878

HFIP-Et2O constrained

FINAL SINGLE POINT ENERGY -1023.343517730692

C	6.67848220506168	-10.69073148998833	-0.93949738773285
C	8.14003654362411	-10.20305871659830	-0.84678529592435
O	6.13155104209740	-10.76008092224776	0.34942194355856
C	6.52007841741618	-12.07648301269001	-1.59430878223481
H	6.15350570806159	-9.98236506054925	-1.58721842033761
F	8.90509418102957	-10.99580494501726	-0.07596720188513
F	8.15680268022777	-8.96926725045808	-0.30013920619636
F	8.72387154988644	-10.11575071491773	-2.05693883734824
F	7.11003742514084	-13.05636695241471	-0.88731445913508
F	7.03293748727703	-12.10297152513420	-2.83923157915582
F	5.21004298644117	-12.37733062185516	-1.68983261745818
H	5.54514803928009	-10.00816466492121	0.49449751045311

H	-8.06837192028043	14.85502171070458	3.62222329319940
H	-11.58882743435081	12.77649939233651	1.10969234594562
H	-10.28427910759036	16.03258110978140	3.46179217470307
H	-12.04022542022132	14.99587963432502	2.20182385733524
C	-8.73198024706948	14.92480672708242	4.48834559540402
C	-10.17373306332983	15.11028727199021	4.05090047437674
O	-10.57005269776776	13.99033923406838	3.26028676035751
C	-12.24885573888424	12.85168303471080	1.97813308893140
C	-11.91856321453558	14.08264801955997	2.80281027760646
H	-8.41390542195167	15.77645994345587	5.09644889015089
H	-13.28109349664985	12.90741891807151	1.62084089033174
H	-8.62036602881671	14.01597218019350	5.08584612566331
H	-12.14135164039756	11.94250758199635	2.57584784711548
H	-10.83647533213919	15.19487640296591	4.92471908289853
H	-12.59425750155781	14.16322471554852	3.66702362937677

References

(1) Huerta-Viga, A.; Shaw, D. J.; Woutersen, S. pH Dependence of the Conformation of Small Peptides Investigated with Two-Dimensional Vibrational Spectroscopy. *J. Phys. Chem. B* **2010**, *114*, 15212–15220.

(2) Cowan, M.; Bruner, B. D.; Huse, N.; Dwyer, J.; Chugh, B.; Nibbering, E.; Elsaesser, T.; Miller, R. Ultrafast memory loss and energy redistribution in the hydrogen bond network of liquid H2O. *Nature* **2005**, *434*, 199–202.

(3) Jansen, T. l. C.; Auer, B.; Yang, M.; Skinner, J. Two-dimensional infrared spectroscopy and ultrafast anisotropy decay of water. *J. Chem. Phys.* **2010**, *132*, 224503.

(4) Yang, M.; Li, F.; Skinner, J. Vibrational energy transfer and anisotropy decay in liquid water: Is the Förster model valid? *J. Chem. Phys.* **2011**, *135*, 164505.

(5) Krevert, C. S.; Gunkel, L.; Haese, C.; Hunger, J. Ion-specific binding of cations to the carboxylate and of anions to the amide of alanylalanine. *Commun. Chem.* **2022**, *5*, 1–10.

(6) Krevert, C. S.; Chavez, D.; Chatterjee, S.; Stelzl, L. S.; Pütz, S.; Roeters, S. J.; Rudzinski, J. F.; Fawzi, N. L.; Girard, M.; Parekh, S. H.; Hunger, J. Liquid-Liquid Phase Separation of the Intrinsically Disordered Domain of the Fused in Sarcoma Protein Results in Substantial Slowing of Hydration Dynamics. *J. Phys. Chem. Lett.* **2023**, *14*, 11224–11234.

(7) Hamm, P.; Zanni, M. T. *Concepts and Methods of 2D Infrared Experiments*; Cambridge University Press: Oxford, UK, 2011.

(8) Blackham, D. V.; Pollard, R. D. An improved technique for permittivity measurements using a coaxial probe. *IEEE Trans. Instrum. Meas.* **1997**, *46*, 1093–1099.

(9) Kaatze, U. Measuring the dielectric properties of materials. Ninety-year development from low-frequency techniques to broadband spectroscopy and high-frequency imaging. *Meas. Sci. Technol.* **2012**, *24*, 012005.

(10) Ensing, W.; Hunger, J.; Ottosson, N.; Bakker, H. On the orientational mobility of water molecules in proton and sodium terminated nafion membranes. *J. Phys. Chem. C* **2013**, *117*, 12930–12935.

(11) Balos, V.; Kim, H.; Bonn, M.; Hunger, J. Dissecting Hofmeister effects: direct anion–amide interactions are weaker than cation–amide binding. *Angew. Chem. Int. Ed.* **2016**, *55*, 8125–8128.

(12) Schrödle, S.; Hefter, G.; Kunz, W.; Buchner, R. Effects of Nonionic Surfactant C12E_5 on the Cooperative Dynamics of Water. *Langmuir* **2006**, *22*, 924–932.

(13) Neese, F.; Wennmohs, F.; Becker, U.; Riplinger, C. The ORCA quantum chemistry program package. *J. Chem. Phys.* **2020**, *152*, 224108.

(14) Becke, A. D. Density-functional thermochemistry. III. The role of exact exchange. *J. Chem. Phys.* **1993**, *98*, 5648–5652.

(15) Lee, C.; Yang, W.; Parr, R. G. Development of the Colle-Salvetti correlation-energy formula into a functional of the electron density. *Phys. Rev. B* **1988**, *37*, 785.

(16) Vosko, H.; Wilk, L.; Nusair, M. Accurate spin-dependent electron liquid correlation energies for local spin density calculations: a critical analysis. *Can. J. Phys.* **1980**, *58*, 1200–1211.

(17) Stephens, J.; Devlin, F.; Chabalowski, C.; Frisch, M. Ab Initio Calculation of Vibrational Absorption and Circular Dichroism Spectra Using Density Functional Force Fields. *J. Phys. Chem.* **1994**, *98*, 11623–11627.

(18) Krishnan, R.; Binkley, J. S.; Seeger, R.; Pople, J. A. Self-consistent molecular orbital methods. XX. A basis set for correlated wave functions. *J. Chem. Phys.* **1980**, *72*, 650–654.

(19) Klamt, A.; ü rmann, G. S. COSMO: a new approach to dielectric screening in solvents with explicit expressions for the screening energy and its gradient. *J. Chem. Soc., Perkin Trans.* **1993**, *2*, 799–805.

(20) Andzelm, J.; lmel, C. K.; Klamt, A. Incorporation of solvent effects into density functional calculations of molecular energies and geometries. *J. Chem. Phys.* **1995**, *103*, 9312–9320.

(21) Barone, V.; Cossi, M. Quantum Calculation of Molecular Energies and Energy Gradients in Solution by a Conductor Solvent Model. *J. Phys. Chem. A* **1998**, *102*, 1995–2001.

(22) Cossi, M.; Rega, N.; Scalmani, G.; Barone, V. Energies, Structures, and Electronic Properties of Molecules in Solution with the C-PCM Solvation Model. *J. Comput. Chem.* **2003**, *24*, 669–681.

(23) Schaal, H.; Häber, T.; Suhm, M. A. Hydrogen bonding in 2-propanol. The effect of fluorination. *J. Phys. Chem. A* **2000**, *104*, 265–274.

(24) Czarnik-Matusewicz, B.; Pilorz, S.; Bieńko, D.; Michalska, D. Molecular and electronic structures, infrared spectra, and vibrational assignment for ap and sc conformers of hexafluoro-iso-propanol. *Vib. Spectrosc.* **2008**, *47*, 44–52.

(25) Rezus, Y.; Bakker, H. Strong slowing down of water reorientation in mixtures of water and tetramethylurea. *J. Phys. Chem. A* **2008**, *112*, 2355–2361.

(26) Cota, R.; van Dam, E. P.; Woutersen, S.; Bakker, H. J. Slowing Down of the Molecular Reorientation of Water in Concentrated Alkaline Solutions. *J. Phys. Chem. B* **2020**, *124*, 8309–8316.

(27) Shinokita, K.; Cunha, A. V.; Jansen, T. L.; Pshenichnikov, M. S. Hydrogen bond dynamics in bulk alcohols. *J. Chem. Phys.* **2015**, *142*, 212450.

(28) Feng, C. J.; Tokmakoff, A. The dynamics of peptide-water interactions in dialanine: An ultrafast amide i 2D IR and computational spectroscopy study. *J. Chem. Phys.* **2017**, *147*, 085101.

(29) Ehrhard, A. A.; Gunkel, L.; Jäger, S.; Sell, A. C.; Nagata, Y.; Hunger, J. Elucidating Conformation and Hydrogen-Bonding Motifs of Reactive Thiourea Intermediates. *ACS Catalysis* **2022**, *12*, 12689–12700.

(30) Asbury, J. B.; Steinel, T.; Fayer, M. D. Vibrational echo correlation spectroscopy probes of hydrogen bond dynamics in water and methanol. *Journal of Luminescence* **2004**, *107*, 271–286.

(31) Knop, S.; Jansen, T. L. C.; Lindner, J.; Vöhringer, P. On the nature of OH-stretching vibrations in hydrogen-bonded chains: Pump frequency dependent vibrational lifetime. *Phys. Chem. Chem. Phys.* **2011**, *13*, 4641–4650.

(32) Ehrhard, A. A.; Jäger, S.; Malm, C.; Basaran, S.; Hunger, J. CF3-groups critically enhance the binding of thiourea catalysts to ketones – a NMR and FT-IR study. *J. Mol. Liq.* **2019**, *296*, 111829.
